# CDK4/6 inhibitors induce replication stress to cause long‐term cell cycle withdrawal

**DOI:** 10.15252/embj.2021108599

**Published:** 2022-01-17

**Authors:** Lisa Crozier, Reece Foy, Brandon L Mouery, Robert H Whitaker, Andrea Corno, Christos Spanos, Tony Ly, Jeanette Gowen Cook, Adrian T Saurin

**Affiliations:** ^1^ Division of Cellular and Systems Medicine Jacqui Wood Cancer Centre School of Medicine University of Dundee Dundee UK; ^2^ Curriculum in Genetics and Molecular Biology University of North Carolina at Chapel Hill Chapel Hill NC USA; ^3^ Department of Biochemistry and Biophysics University of North Carolina at Chapel Hill Chapel Hill NC USA; ^4^ Wellcome Trust Centre for Cell Biology University of Edinburgh Edinburgh UK; ^5^ Present address: Centre for Gene Regulation and Expression School of Life Sciences University of Dundee Dundee UK

**Keywords:** CDK6, cyclin‐dependent kinase, Palbociclib, replication stress, senescence, Cancer, Cell Cycle, DNA Replication, Recombination & Repair

## Abstract

CDK4/6 inhibitors arrest the cell cycle in G1‐phase. They are approved to treat breast cancer and are also undergoing clinical trials against a range of other tumour types. To facilitate these efforts, it is important to understand why a cytostatic arrest in G1 causes long‐lasting effects on tumour growth. Here, we demonstrate that a prolonged G1 arrest following CDK4/6 inhibition downregulates replisome components and impairs origin licencing. Upon release from that arrest, many cells fail to complete DNA replication and exit the cell cycle in a p53‐dependent manner. If cells fail to withdraw from the cell cycle following DNA replication problems, they enter mitosis and missegregate chromosomes causing excessive DNA damage, which further limits their proliferative potential. These effects are observed in a range of tumour types, including breast cancer, implying that genotoxic stress is a common outcome of CDK4/6 inhibition. This unanticipated ability of CDK4/6 inhibitors to induce DNA damage now provides a rationale to better predict responsive tumour types and effective combination therapies, as demonstrated by the fact that CDK4/6 inhibition induces sensitivity to chemotherapeutics that also cause replication stress.

## Graphical abstract

Genotoxic effects may underlie the long‐lasting proliferative arrest triggered by anti‐cancer drugs that cause G1 phase pausing.

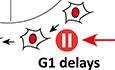

## Introduction

Cyclin‐dependent kinases 4 and 6 (CDK4/6) phosphorylate the retinoblastoma protein (Rb) to relieve repression of E2F‐dependent genes and allow progression from G1‐ into S‐phase. Three structurally distinct CDK4/6 inhibitors have recently been licenced for breast cancer treatment: palbociclib, ribociclib and abemaciclib (Knudsen & Witkiewicz, 2017; Alvarez‐Fernandez & Malumbres, 2020). Unlike other cell cycle inhibitors, these agents are generally well‐tolerated and have demonstrated remarkable efficacy in treating hormone receptor‐positive/human epidermal growth factor 2‐negative (HR+/HER2‐) metastatic breast cancer (Dickler *et al*, 2017; Turner *et al*, 2018; Im *et al*, 2019; Johnston *et al*, 2019). Comparisons with standard‐of‐care chemotherapy have given weight to the notion that CDK4/6 inhibitors may be able to replace conventional chemotherapy in this cancer subtype, which represents the majority of metastatic breast cancers (Giuliano *et al*, 2019; Nasrazadani & Brufsky, 2019; Tolaney *et al*, 2020).

There is also a wealth of preclinical evidence that CDK4/6 inhibitors display broad activity against a wide range of other tumour types (for reviews see (Asghar *et al*, 2015; O'Leary *et al*, 2016; Du *et al*, 2020)). This is supported by preliminary clinical data suggesting that these inhibitors may be beneficial for treating non‐small cell lung cancer, melanoma, head and neck squamous cell carcinoma, mantle cell lymphoma, triple‐negative breast cancer and acute myeloid leukaemia (Patnaik *et al*, 2016; Asghar *et al*, 2017; Adkins *et al*, 2019; Tan *et al*, 2019; Du *et al*, 2020; Morschhauser *et al*, 2020). Currently, there are at least 18 different CDK4/6 inhibitors being tested in over 100 clinical trials against various tumour types (for reviews see (Asghar *et al*, 2015; O'Leary *et al*, 2016; Klein *et al*, 2018; Alvarez‐Fernandez & Malumbres, 2020; Yuan *et al*, 2021)). The hope is that these targeted cell cycle inhibitors may be widely applicable for cancer treatment, perhaps offering an alternative to the non‐targeted, and considerably more toxic, DNA damaging agents or microtubule poisons that are currently in widespread clinical use.

To facilitate these efforts, there is an urgent need to identify biomarkers and combination treatments that can predict and improve patient outcomes. This requires the characterization of sensitizing events that can either: (i) enhance the ability of CDK4/6 inhibitors to arrest the cell cycle in G1, or (ii) improve long‐term outcomes following this G1 arrest. Although various genetic backgrounds and drug treatments are known to sensitize the CyclinD‐CDK4/6‐Rb pathway and promote an efficient G1 arrest (Gong *et al*, 2017; Knudsen & Witkiewicz, 2017; Vijayaraghavan *et al*, 2017; Goel *et al*, 2018; Xue *et al*, 2019a, 2019b; Alvarez‐Fernandez & Malumbres, 2020), relatively little is known about sensitizing events that could improve long‐term growth suppression following this arrest. The problem is that there is no clear consensus for why a G1 arrest, which is essentially cytostatic, can produce durable effects in patients. There are many different potential explanations, including that CDK4/6 inhibition can induce senescence, apoptosis, metabolic reprogramming and/or anti‐tumour immunity (for reviews see (Goel *et al*, 2018; Klein *et al*, 2018)), but whether a common event underlies these different outcomes is unclear. There is good evidence that some of the long‐term outcomes are linked, in particular, senescent cells can secrete a variety of factors that engage the immune system (Xue *et al*, 2007; Krizhanovsky *et al*, 2008; Kang *et al*, 2011; Acosta *et al*, 2013), and this senescence phenotype contributes to the ability of CDK4/6 inhibition to sensitize tumours to immune checkpoint blockade (Jerby‐Arnon *et al*, 2018; Ruscetti *et al*, 2018, 2020). It is therefore critical to determine how and why G1‐arrested cells eventually become senescent because this may help to inform ongoing clinical trials assessing CDK4/6 inhibition alongside immunotherapy (currently 14 trials in eight different cancer types (Wagner & Gil, 2020)).

Senescence is a state of irreversible cell cycle exit induced by stress, typically DNA damage or oxidative stress (Mijit *et al*, 2020). A crucial question therefore concerns the nature of the stress that leads to senescence following CDK4/6 inhibition. Unfortunately, although senescence has been demonstrated in a variety of different studies (for recent review see (Wagner & Gil, 2020)), only two of these studies report a source for the stress. In both cases, senescence is believed to be induced by ROS generated during a G1 arrest (Vijayaraghavan *et al*, 2017), perhaps as a result of FOXM1 destabilization (Anders *et al*, 2011). There have been more attempts to characterize the mediator(s) of the subsequent senescent response, but the answers here have been varied, including a dependence on ATRX (Kovatcheva *et al*, 2015, 2017), proteasome activation (Miettinen *et al*, 2018), mTOR activation (Leontieva & Blagosklonny, 2013) or mTOR inhibition (Yoshida *et al*, 2016). This variability may reflect inherent differences between genomically diverse cancer lines. Alternatively, it may be due to inconsistent treatment protocols (drug type, dose, duration of exposure and length of washout) or the reliance on fixed endpoints that can only indirectly measure senescence (Sharpless & Sherr, 2015).

To overcome these problems, we initially elected to use a non‐transformed near‐diploid telomerase‐immortalized hTert‐RPE1 cell line expressing a FUCCI cell cycle reporter to track the fate of single cells over time following CDK4/6 inhibition. We compared all currently licenced CDK4/6 inhibitors over a range of treatment protocols to address one key unexplained question: why do these inhibitors cause long‐term cell cycle exit? Our results demonstrate that a prolonged G1 arrest is associated with the downregulation of replisome components, including the MCM complex, which causes reduced origin licencing, replication stress, p53‐p21 activation and long‐term cell cycle withdrawal. Similar effects are observed in a range of cancer lines, which demonstrates that the induction of genotoxic stress is a common outcome of targeted CDK4/6 inhibition. These findings have considerable implications for the identification of sensitive/resistant tumour types, for the design of effective combination treatments and drug dosing schedules, and for the efforts to use CDK4/6 inhibitors to sensitize tumours to immune checkpoint blockade.

## Results

**Figure 1:**
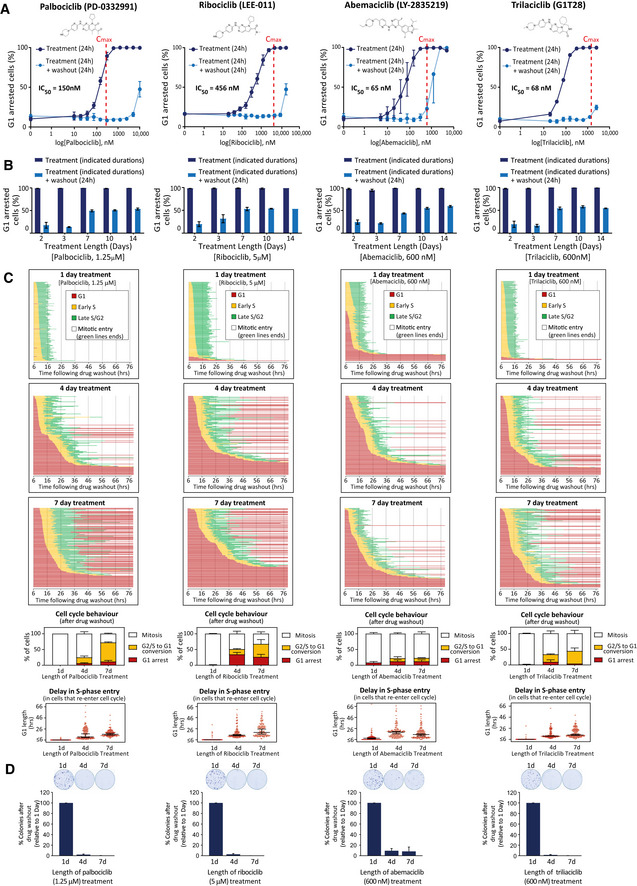
A prolonged G1 arrest following CDK4/6 inhibition in RPE1 cells causes defects in the next cell cycle Top panel displays structure of each CDK4/6 inhibitor tested. Bottom panel shows dose–response curves with these inhibitors displaying percentage of G1‐arrested RPE1‐FUCCI cells. To obtain dose–response curves, the number of mKO‐Cdt1‐positive (G1‐arrested) cells was calculated following 24 h drug addition (dark blue solid lines) or 24 h after subsequent drug washout (light blue dotted lines). *C*_max_ values observed in patients (taken from (He *et al*, 2017; Klein *et al*, 2018)) are represented on each graph with red dotted lines. Graphs display mean data ± SEM from three experiments, with at least 500 cells counted per condition per experiment.Percentage of G1‐arrested RPE1‐FUCCI cells, calculated as in panel (A), but using a fixed concentration of CDK4/6 inhibitor for different durations of time, as indicated. Note, this is a fixed assay that quantifies the percentage of G1‐arrested cells prior to, or 24 h following, CDK4/6 inhibitor washout. Each bar displays mean data + SEM from three experiments, with at least 500 cells counted per condition per experiment.Cell cycle profile of individual RPE1‐FUCCI cells (each bar represents one cell) after washout from 1 (top panel), 4 (middle panel) or 7 (bottom panel) days of treatment with CDK4/6 inhibitor, at the indicated doses (same concentration used in panel (B)). STLC (10 μM) was added to prevent progression past the first mitosis. Fifty cells were analysed at random for each repeat and three experimental repeats are displayed (150 cells total). Underneath the single‐cell profiles are quantifications of the observed cell cycle defects and G1 durations. Note, G1 length is estimated by mKO‐Cdt1 expression, and G1 lengths of less than 6 h could not be quantified since movies were only started after the 6 h drug washout period. These are indicated on the graphs as ≤ 6 h. Bar graphs show mean + SD. In the violin plots, horizontal lines display the median, and error bars show 95% confidence intervals.Representative images and quantification of colony forming assays of RPE1 cells treated with CDK4/6 inhibitor for 1, 4 or 7 days and then grown at low density without inhibitor for 10 days. Bar displays mean data + SEM from three experiments.

**Figure EV1:**
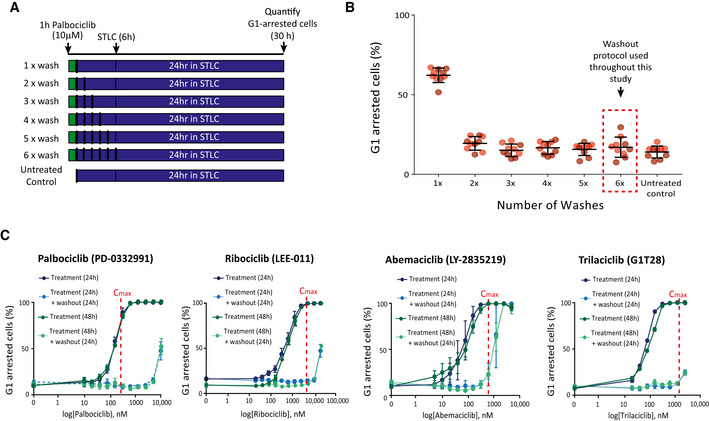
Effect of drug washout protocol or inhibiting CDK4/6 for 48 h on the reversibility of the G1 arrest in RPE1 cells Schematic showing different washout protocols tested to ensure washout of high‐dose (10 μM) palbociclib. RPE1‐FUCCI cells were treated for 1 h with 10 μM palbociclib and subsequently washed out 1–6 times, with 1 h equilibration periods interspersed between washes. STLC (10 μM) was then added to arrest cells in mitosis before quantifying the amount of mKO‐Cdt1‐positive, G1‐arrested cells 24 h later.Quantification of the G1‐arrested cells following the washout protocol described in (A). Graphs display the mean data ± SD from at least 500 cells counted per condition per experiments for two experimental repeats. Note, the points represent five different positions that were imaged per condition per experiment and the different coloured dots represent the two separate experiments.Percentage of G1‐arrested RPE1‐FUCCI cells after treatment for 48 h with different CDK4/6 inhibitors (dark green solid lines) or 24 h after subsequent drug washout (light green dotted lines). The data are overlaid with 24 h arrest data from Fig 1A (blue lines) to allow comparison. Vertical red dotted lines indicate *C*_max_ values observed in patients (taken from (He *et al*, 2017; Klein *et al*, 2018)). Graphs display mean data ± SEM from three experiments, with at least 500 cells counted per condition per experiment.

We first quantified the fraction of G1‐arrested RPE1‐FUCCI cells (Krenning *et al*, 2014) following 24 h treatment with four structurally distinct CDK4/6 inhibitors: palbociclib (PD‐0332991), ribociclib (LEE‐011), abemaciclib (LY‐2835219), which are licensed for breast cancer treatment, and trilaciclib (G1T28), which was recently approved to reduce chemotherapy‐induced myelosuppression in small‐cell lung cancer (Dhillon, 2021). The dose–response curves for all inhibitors demonstrate a penetrant arrest at the clinically relevant peak plasma concentrations (*C*_max_) observed in patients (He *et al*, 2017; Klein *et al*, 2018) (Fig 1A). Note, RPE1 cells are exquisitely sensitive to these compounds since the IC50s for palbociclib and abemaciclib (150 and 65 nM, respectively) were comparable to the IC50 values reported in the most sensitive cell type from a panel of 560 tumour lines: 130 nM palbociclib in MDA‐MB‐175‐VII (breast cancer) and 60 nM abemaciclib in JeKo‐1 (mantle cell lymphoma) (Gong *et al*, 2017). At approximate *C*_max_ concentrations or lower, the G1 arrest was fully reversible within 24 h of drug washout, although at higher concentrations this reversibility is compromised for all drugs (Fig 1A). Note that we used an extensive washout protocol to ensure that persistent arrest is due to effects on the cell cycle and not incomplete drug washout (Fig EV1A and B; this protocol was used in all subsequent washout experiments) and cells were always plated at a low density to prevent G1 arrest due to contact inhibition (Mendonsa *et al*, 2018) (see Materials and Methods). The irreversible effects at higher drug concentrations are likely to represent off‐target effects, as reported previously for palbociclib at ≥ 5 μM concentrations (Vijayaraghavan *et al*, 2017). In general, abemaciclib displayed the narrowest concentration range in which to achieve an efficient arrest that remained reversible, as noted recently by others (Trotter & Hagan, 2020). The fact that abemaciclib is uniquely able to induce irreversible effects at approximate *C*_max_ concentrations may help to explain the unique toxicity profile associated with this drug (Marra & Curigliano, 2019).

Increasing the duration of drug exposure to 48 h produced almost identical dose–response curves, indicating that after 2 days of treatment all drugs induced a similarly reversible G1 arrest (Fig EV1C). We next used the minimal dose of each drug required to produce a fully penetrant G1 arrest for 24 h and assessed the ability of this dose to induce a prolonged arrest for up to 14 days. Figure 1B demonstrates that all drugs can hold a full G1 arrest for up to 2 weeks. However, upon release from a prolonged arrest (> 3 days), we observed an increase in the fraction of cells remaining in G1. Therefore, CDK4/6 inhibition can induce a penetrant and reversible cell cycle arrest in RPE1 cells, but this reversibility is compromised when drug treatment persists for longer than 3 days.

This analysis only provides the cell cycle status at a single time point (24 h) following drug washout. Therefore, to examine this phenotype more closely, we performed live single‐cell fate analysis using RPE1‐FUCCI cells during the first cell cycle after washout from different durations of CDK4/6 inhibitor treatment (Fig 1C) (Sakaue‐Sawano *et al*, 2008). Using this approach, we observed two striking phenotypes that appeared specifically following release from prolonged drug exposure. Firstly, the length of time individual cells took to exit G1 and enter S‐phase following drug washout increased: most cells took many hours to exit G1 and a small fraction of cells failed to exit G1 at all within the 3‐day imaging period. This is suggestive of a deep G1 arrest, which may become irreversible in a subset of cells. Secondly, following washout from 4‐ and 7‐day treatments, many cells that entered S‐phase failed to reach mitosis and instead reverted back into a G1‐like state: green bars turning red (G1) instead of white (mitosis) in Fig 1C. This was not due to depletion of nutrients in the media since it was unaffected by replenishing the media daily (Appendix Fig S1). Therefore, prolonged arrest with CDK4/6 inhibitors induces a deep G1 arrest, and many cells that exit from this arrest fail to complete the next cell cycle. Colony forming assays demonstrated that these effects are associated with long‐term inhibition of cell proliferation (Fig 1D).

**Figure 2:**
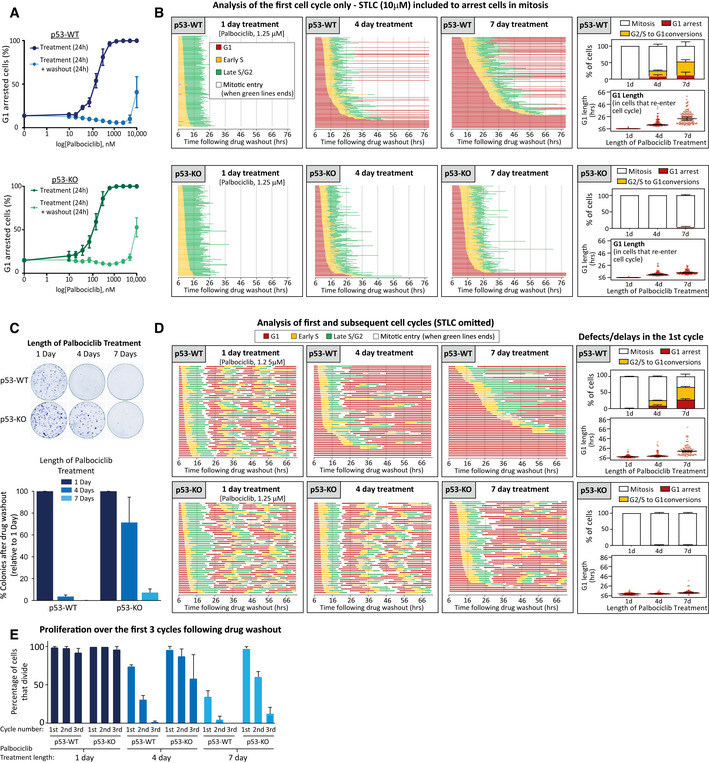
p53 loss restores cell cycle progression and enhances long‐term proliferation following prolonged CDK4/6 inhibition in RPE1 cells Dose–response curves displaying the percentage of G1‐arrested p53‐WT (blue) or KO (green) RPE1‐FUCCI cells following 24 h incubation with palbociclib (dark solid lines) or 24 h after subsequent washout (light dotted lines). Graphs display mean data ± SEM from three experiments, with at least 500 cells counted per condition per experiment.Cell cycle profile of individual p53‐WT or KO RPE1‐FUCCI cells (each bar represents one cell) after washout from 1, 4 or 7 days of treatment with palbociclib (1.25 μM). STLC (10 μM) was added to prevent progression past the first mitosis. Fifty cells were analysed at random for each repeat and three experimental repeats are displayed (150 cells total). The right‐side panels show quantifications of cell cycle defects and G1 lengths from the displayed single‐cell profile plots. Note, G1 length is estimated by mKO‐Cdt1 expression, and G1 lengths of less than 6 h could not be quantified since movies were only stated after the 6 h drug washout period. These are indicated on the graphs as < 6 h. Bar graphs show mean + SD. In the violin plots, horizontal lines display the median, and error bars show 95% confidence intervals.Representative images and quantifications of colony forming assays in p53‐WT or KO RPE1 cells treated with palbociclib (1.25 μM) for 1, 4 or 7 days and then grown at low density without inhibitor for 10 days. Each bar displays mean data + SEM from three experiments.Cell cycle profile of individual p53‐WT or KO RPE1‐FUCCI cells to analyse multiple rounds of division following washout from 1, 4 or 7 days of treatment with palbociclib (1.25 μM). FUCCI profiles show 50 cells analysed at random from one experiment, which is representative of three experimental repeats. The right‐side panels show quantifications of cell cycle defects and G1 lengths from the three experimental repeats. Note, G1 length is estimated by mKO‐Cdt1 expression, and G1 lengths of less than 6 h could not be quantified since movies were only stated after the 6 h drug washout period. These are indicated on the graphs as < 6 h. Bar graphs show mean + SD. In the violin plots, horizontal lines display the median, and error bars show 95% confidence intervals.Quantification of cell cycle profiles from cells treated as in (D). Graph shows the mean percentage of cells + SD that divide at each round of division, with 150 cells analysed in total from three experimental repeats.

The reversion of cells from S‐phase/G2 back into G1 has previously been associated with a p53‐dependent senescent response (Johmura *et al*, 2014; Krenning *et al*, 2014; Gire & Dulic, 2015). To explore the role of p53 in these phenotypes, we performed similar cell fate analysis in p53‐WT and p53‐KO RPE1‐FUCCI cells, generated by CRISPR/Cas9‐mediated gene editing (Appendix Fig S2). Figure 2A demonstrates that 24 h palbociclib treatment induced a dose‐dependent reversible G1 arrest that was indistinguishable between p53‐WT and KO cells. Although knockout of p53 did not affect the efficiency of a palbociclib‐induced arrest, it did produce a striking effect on the phenotypes observed following washout from prolonged exposure to 1.25 μM palbociclib (Fig 2B). Firstly, the delay in S‐phase entry following drug washout was less pronounced and fewer cells remained arrested in G1 for the duration of the movie. Secondly, the conversions from S‐phase/G2 into G1 were completely abrogated.

To determine whether these cell cycle defects were associated with a reduction in long‐term proliferation, we performed colony forming assays under identical conditions. Figure 2C shows that 4 days of palbociclib treatment is sufficient to dramatically reduce colony forming potential in p53‐WT cells, whereas 7 days of palbociclib is required to cause a similar reduction in p53‐KO cells. We were struck by two major differences between the long‐term proliferation data and the cell cycle analysis (Fig 2B and C). Firstly, 4 days of palbociclib treatment induced relatively few cell cycle withdrawals in p53‐WT cells (16% S/G2 to G1 conversions) but this was associated with a strong reduction in long‐term proliferation. Secondly, although removal of p53 allowed all cells to progress into mitosis following 4‐ or 7‐day palbociclib treatment (Fig 2B, lower panels), p53 loss could only restore long‐term proliferation in the 4‐day treatment group (Fig 2C). Our FUCCI analysis only allowed quantification of the first cell cycle following drug release because cells were released from palbociclib in the presence of the Eg5 inhibitor *S*‐trityl‐l‐cysteine (STLC) to block cells in mitosis (DeBonis *et al*, 2004). To analyse additional cell cycles after release, we performed FUCCI analysis without STLC and analysed the first 3 days of proliferation following palbociclib release. This demonstrated that although most p53‐WT and KO cells were able to complete the first cell cycle following washout from 4‐day palbociclib treatment, only the p53‐KO cells were able to continue proliferating at a normal rate during subsequent cell cycles (Fig 2D and E), consistent with the difference in colony forming potential observed in the 4‐day treatment groups (Fig 2C). The proliferative ability of p53‐KO cells was compromised after 7 days of palbociclib treatment, however, since considerably fewer mitotic events were apparent during the first 3 days following drug washout. This pattern also correlated with the reduction in long‐term proliferation in this condition (Fig 2C). In general, cell cycle behaviour over the first 3 days was predictive of long‐term proliferative potential, with only the normally dividing cells (i.e. approx. 24 h cell cycles) able to form visible colonies (Fig 2C and D). Therefore, CDK4/6 inhibition for longer than 3 days causes defects in subsequent cell cycles which restricts long‐term proliferative potential. This effect can be partially rescued by knockout of p53 which allows cells to tolerate an extended window of palbociclib treatment before they begin to exit the cell cycle. This may help to explain why p53 loss is associated with resistance to CDK4/6 inhibition in patients (Patnaik *et al*, 2016; Wander *et al*, 2020).

**Figure 3:**
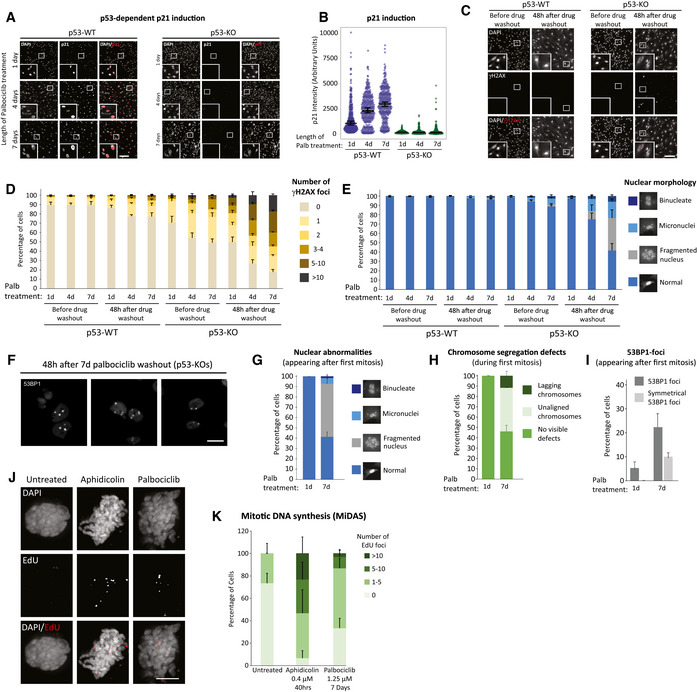
Prolonged CDK4/6 inhibition in RPE1 cells induces replication stress and p53‐dependent cell cycle withdrawal ARepresentative immunofluorescence images of p21 levels in p53‐WT or KO RPE1 cells, 48 h after release from 1, 4 or 7 days palbociclib (1.25 μM) treatment. Zoom inserts are 3× magnification of the indicated regions. Scale bars = 250 μM.BQuantification of p21 intensities in cells treated as in panel (A). At least 100 cells were analysed per experiment and graph shows data from three experimental repeats. Violin plots display the variation in intensities between individual cells. Horizontal lines display the median, and error bars show 95% confidence intervals.CImmunofluorescence images of DAPI and γH2AX staining in p53‐WT or KO RPE1 cells either before or 48 h after release from a 7‐day treatment with palbociclib (1.25 μM). Scale bar = 250 μM, zoom inserts = 3× magnification of highlighted regions.D, EQuantification of nuclear morphologies (D) and γH2AX‐positive DNA damage foci (E) following palbociclib (1.25 μM) treatment in p53‐WT and KO RPE1 cells. Cells were treated for 1, 4 or 7 days and then analysed before or after drug washout for 48 h. A total of 100 cells (nuclear morphology) or 50 cells (γH2AX foci) were scored per condition per experiment, and bar graphs represent mean data + SEM from six experiments.FImmunofluorescence images showing symmetrical 53BP1 staining following mitotic exit in p53‐KO cells after release from 7 days of palbociclib arrest. Three separate examples are displayed. Scale bar = 25 μM.G–IAnalysis of chromosome segregation errors and DNA damage during the first mitosis in GFP‐53BP1/H2B‐RFP RPE1 cells after release of from a 1‐ or 7‐day palbociclib (1.25 μM) arrest. Quantified from the same movies are nuclear morphology after mitosis (G), chromosome segregation defects during mitosis (H) and appearance of 53BP1 foci after mitosis (I). A total of 54 cells (1 day) or 80 cells (7 days) were analysed in total from two experiments. Errors bars display SD.J, KRepresentative immunofluorescence images (J) and quantifications (K) of mitotic DNA replication assays (MiDAS) in p53‐KO RPE1 cells released from 7 days of palbociclib (1.25 μM) treatment or following 0.4uM aphidicolin treatment for 40 h. EdU foci were quantified in nocodazole‐arrested cells. Scale bar = 5 μM, zoom inserts = 3× magnification of highlighted areas. Ten cells were analysed per experiment and the bar chart shows the mean + SEM from three experimental repeats.

**Figure EV2:**
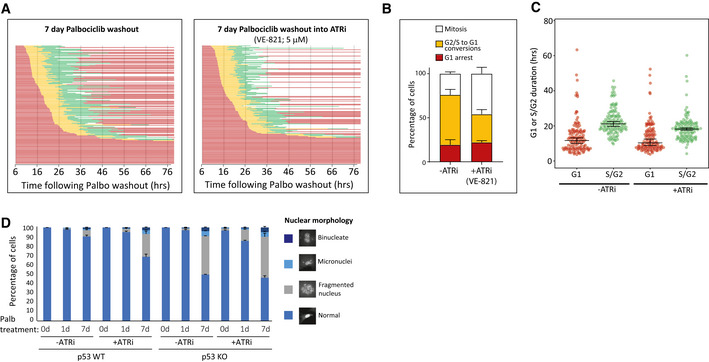
Effect of ATR inhibition on the response of RPE1 cells to CDK4/6 inhibition ASingle‐cell cycle profiles quantified immediately following washout from 7 days 1.25 µM Palbociclib treatment. After washout, cells were cultured in the absence or presence of the ATR inhibitor, VE‐821 (5 µM). STLC was also added at drug washout to allow analysis of just the first cell cycle. Each bar represents an individual cell, and graphs show the data from three experimental repeats (150 cells analysed in total).B, CQuantifications of the cell cycle defects (B) and G1 or S/G2 durations (C) from the single‐cell profiles shown in (A). Note, G1 length is estimated by the duration of mKO‐Cdt1 expression, and S/G2 by the time from AG‐Geminin expression until mitotic entry. Only cells that re‐enter the cell cycle were included in this quantification (G1‐arrested cells excluded). Bars graphs in (B) display mean + SD from three experimental repeats, violin plots in (C) display the variation in G1 and S/G2 length between individual cells, with horizontal lines indicating the median, and error bars representing 95% confidence intervals.DQuantification of the nuclear morphologies following palbociclib (1.25 μM) treatment in p53‐WT and KO RPE1 cells. Cells were treated for 0, 1 or 7 days and before washout for 48 h (± ATR inhibition with 5 µM VE‐821). Nuclear morphologies of 100 cells were counted per condition and per experiment, and bar graphs represent mean data + SEM from three experiments.

We next investigated the reason for cell cycle withdrawal following CDK4/6 inhibition. The ability of p53 to induce cell cycle exit from G2 has previously been linked to p21 induction (Johmura *et al*, 2014; Krenning *et al*, 2014), therefore we analysed p21 levels following CDK4/6 inhibition. In p53‐WT cells, we observed that prolonged CDK4/6 inhibition is associated with a strong induction of p21 both during and following the G1 arrest (Fig 3A and B, and Appendix Fig S3). p21 induction was absent in p53‐KO cells, as expected, which is consistent with the inability of these cells to exit the cell cycle from G2. The lack of p53‐induced p21 had dramatic consequences because instead of withdrawing from the cell cycle, p53‐KO cells underwent a catastrophic mitosis that produced excessive DNA damage, as judged by nuclear morphology and γH2AX staining (Fig 3C–E). This also caused the appearance of symmetrical 53BP1 nuclear bodies after mitosis, a phenotype that results from the segregation of incompletely replicated chromosomes (Fig 3F) (Harrigan *et al*, 2011; Lukas *et al*, 2011). Live cell imaging of GFP‐53BP1/H2B‐RFP p53‐KO RPE1 cells confirmed that DNA damage specifically appeared after an abnormal mitosis, and this was frequently associated with the segregation of unaligned or lagging chromosomes (Fig 3G–I). Examples of the abnormal divisions can be seen in Movie EV1 (7 days palbociclib washout) in comparison to Movie EV2 (1‐day palbociclib washout). Cells have intrinsic mechanisms to either replicate or resolve incompletely replicated DNA during mitosis, but these systems can be overwhelmed under conditions of replication stress to cause DNA strand breaks during mitosis (Minocherhomji *et al*, 2015; Nielsen *et al*, 2015; Pedersen *et al*, 2015; Moreno *et al*, 2016; Gemble *et al*, 2020). To examine whether DNA replication was indeed ongoing during mitosis, we performed mitotic DNA replication assays by examining EdU incorporation in mitotic cells 15 min after washout from the CDK1 inhibitor RO‐3306 (Vassilev *et al*, 2006). Aphidicolin treatment, a well‐known inducer of replication stress, was sufficient to elevate the levels of mitotic DNA replication in p53‐KO cells, as expected (Minocherhomji *et al*, 2015) (Fig 3J and K). This increase in mitotic DNA replication was also observed after release from a prolonged palbociclib arrest (Fig 3J and K), consistent with the notion that DNA replication is also perturbed in these cells. Note, very few p53‐WT cells enter mitosis after prolonged palbociclib release, which we hypothesize is due to a combination of p53‐dependent cell cycle withdrawal (Fig 2B) and an intact ATR‐dependent checkpoint that prevents mitotic entry until DNA replication is complete (Saldivar *et al*, 2018). In support of the latter, inhibition of ATR directly with VE‐821 (Reaper *et al*, 2011) decreased S/G2 length and increased the number of p53‐WT cells that enter mitosis following release from prolonged CDK4/6 inhibition, and this was also associated with an increase in fragmented nuclei, which are typically produced by chromosome segregation errors (Fig EV2).

In summary, a prolonged palbociclib arrest causes replication stress following release from that arrest. This inhibits long‐term viability by either inducing a p53‐dependent withdrawal from the cell cycle or, in the absence of p53, by causing cells to undergo a catastrophic mitosis resulting in DNA damage as under‐replicated chromosomes are segregated. If this damage is too excessive, then long‐term proliferation is still affected, as observed following release from a 7‐day palbociclib arrest (Figs 2C and D and 3C–E). If the replication stress is milder, for example, following 4‐day palbociclib arrest, then cells can progress through mitosis but frequently arrest in a p53‐dependent manner in the subsequent G1 (Fig 2B–D). This is consistent with the previous observations that mild replication stress causes a p21‐dependent arrest in the subsequent G1 (Arora *et al*, 2017; Barr *et al*, 2017).

**Figure 4:**
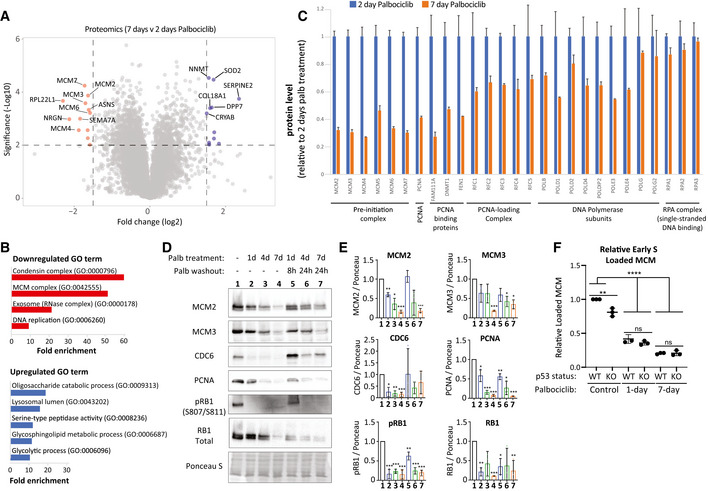
Prolonged G1 arrest following palbociclib treatment in RPE1 cells downregulates replisome components and impairs origin licencing Volcano plot of proteins up or downregulated following prolonged palbociclib (1.25 μM) treatment in RPE1 cells. The top 10 significantly upregulated and downregulated proteins are shown in blue and red respectively.The top up‐ or downregulated Gene Ontology (GO) terms following 7‐day palbociclib (1.25 μM) treatment relative to 2 days of treatment.Quantification of relative change in protein levels of selected replisome components between 2‐day (blue bars) and 7‐day (orange bars) palbociclib (1.25 μM) treatment. Graphs display mean + SD from 2–3 experimental repeats.Representative western blots of whole‐cell lysates from RPE1‐WT cells treated with palbociclib (1.25 μM) for 1, 4 or 7 days, or treated identically, and then washed out for the indicated times to reflect when the majority of cells are in S‐phase (see Fig 1C).Analysis of adjusted relative density from three independent western blot experiments. Bars display mean values ± SD. Significance determined by unpaired Student's *t*‐test comparing treated target protein to asynchronous target control (*< 0.01, ** < 0.001, *** < 0.0001).Quantification of loaded MCM in p53‐WT and KO RPE1 cells treated with palbociclib (1.25uM) for 1 or 7 days followed by drug washout for 8 h after 1 day of arrest or 24 h after 7 days of arrest to capture cells in early S‐phase. To quantify loaded MCM, soluble MCM was pre‐extracted from cells and the amount of the remaining DNA‐loaded MCM was analysed by flow cytometry (see Appendix Fig S4 for representative FACS profiles). DNA content was measured with DAPI, and DNA synthesis was measured using a 30‐min EdU pulse. The amount of DNA‐loaded MCM in early S‐phase cells was compared to untreated control cells. The measured fluorescent intensity of each sample was divided by the background intensity of an identically treated but unstained control. The resulting ratios were normalized to WT control cells. Graphs display mean data ± SD from three experimental repeats. Significance determined by one‐way ANOVA followed by Tukey's multiple comparisons test (**P = 0.001, ****P < 0.0001).

**Figure EV3:**
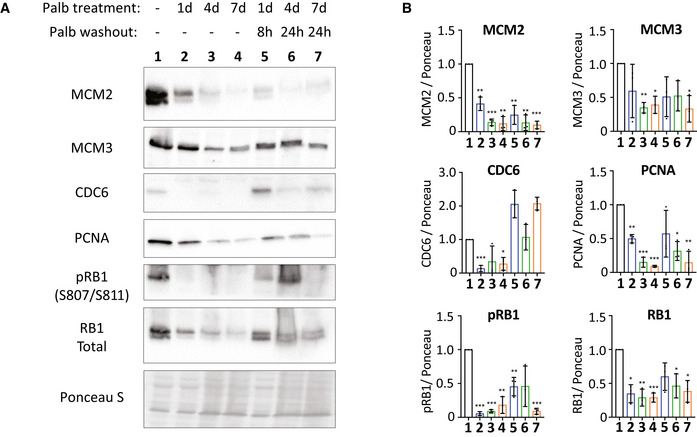
Western blots to characterize the level of replisome proteins in p53‐KO RPE1 cells treated with palbociclib Representative western blots of whole‐cell lysates from p53‐KO RPE1 cells treated with palbociclib (1.25 μM) for 1, 4 or 7 days, or treated identically, and then washed out for the indicated times to reflect when the majority of cells are in S‐phase (see Fig 1C).Analysis of adjusted relative density from three independent western blot experiments. Bars display mean values ± SD. Significance determined by unpaired Student's *t*‐test comparing treated target protein to asynchronous target control (*< 0.01, **< 0.001, ***< 0.0001).

Defects in the cell cycle begin to appear if CDK4/6 inhibitors are applied for longer than 2 days (Figs 1 and EV1C). Therefore, to screen for potential causes of replication stress, we performed a proteomic comparison of cells arrested in palbociclib for 2 or 7 days (Dataset EV1). Of the top 15 most significantly changing proteins, five were members of the MCM2‐7 complex, which licences DNA replication origins and then forms the catalytic core of the CMG (Cdc45, MCM2‐7, GINS) helicase that is responsible for unwinding DNA to allow replication fork progression (Fig 4A and B) (Pellegrini & Costa, 2016). In addition to MCMs, many other components of the core replisome were downregulated by prolonged palbociclib treatment, including the DNA clamp (PCNA), the clamp‐loading complex (RFC1–5) and many accessory factors that bind PCNA (FEN1, DNMT1 and FAM111A). In addition, we observed downregulation of a variety of DNA polymerases along with their accessory subunits (Fig 4C). Western blotting confirmed that the levels of replisome components progressively decreased during a palbociclib arrest and, importantly, remained low after palbociclib washout for 8 or 24 h (Fig 4D and E); time points chosen to capture the majority of cells as they replicate DNA in S‐phase (Fig 1C). In addition to decreasing total MCM protein, palbociclib treatment also reduced the extent of origin licencing after release from the inhibitor, as assessed by the level of chromatin‐bound MCM during early S‐phase (Fig 4F and Appendix Fig S4). Therefore, a palbociclib‐induced G1 arrest both compromises origin licencing during G1 and reduces the concentration of proteins required to assemble functional replication forks during S‐phase. This combination of impairments likely explains why if the G1 arrest is too long, there is a failure in DNA replication after release from that arrest, resulting in either cell cycle exit (p53 proficient) or a catastrophic mitosis with under‐replicated DNA (p53 deficient). Note that the decrease in replisome components and origin licencing was similar in p53‐KO cells (Figs 4D–F and EV3), implying that p53 status primarily defines the response to DNA replication defects.

The ability of CDK4/6 inhibitors to induce genotoxic damage as a result of replication stress has important implications for cancer treatment. Firstly, it suggests that tumour cells with ongoing replication stress may be more sensitive to the long‐term effects of CDK4/6 inhibition. Secondly, it implies that chemotherapeutics that enhance replication stress may sensitize cells to CDK4/6 inhibition. We sought to address these points in a controlled manner using RPE1 cells.

**Figure 5:**
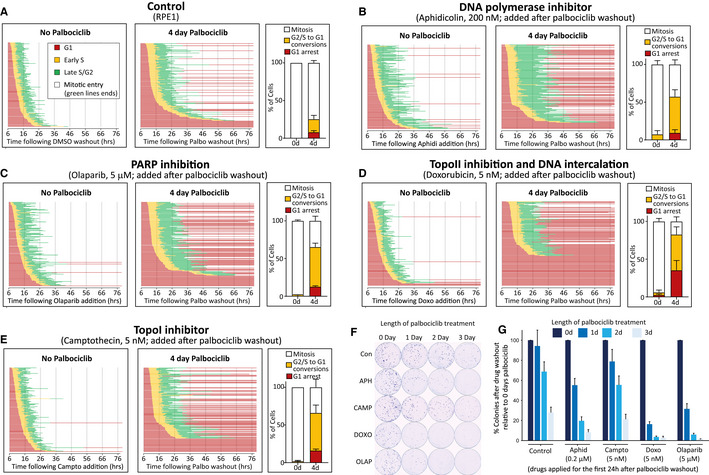
CDK4/6 inhibition sensitizes RPE1 cells to genotoxic chemotherapeutics ACell cycle profile of individual RPE1‐FUCCI cells (each bar represents one cell) after release from 4d treatment with palbociclib (1.25 μM) or DMSO.B–ECell cycle profile of individual RPE1‐FUCCI cells treated as in panel (A), but additionally treated after drug washout with aphidicolin (B), olaparib (C), doxorubicin (D) or camptothecin (E), at indicated concentrations.F, GRepresentative images (F) and quantifications (G) of colony forming assays with RPE1 cells treated with palbociclib (1.25 μM) for indicated times, and then grown at low density without palbociclib for 10 days. DMSO (control) or indicated genotoxic drugs were applied for the first 24 h after palbociclib washout. Each bar displays mean data + SD from four experiments. Data information: All experiments in panels A‐F were run at the same time to allow comparison to the same control (panel (A)). STLC (10 μM) was added in all movies to prevent progression past the first mitosis. In all FUCCI graphs (A–E), 50 cells were analysed at random for each repeat and three experimental repeats are displayed (150 cells total). Bar graphs in (A–E) show mean + SD.

We therefore used a low dose of aphidicolin to partially inhibit DNA polymerases and induce replication stress directly. Figure 5A and B shows that while this did not have a strong effect on cell cycle progression when given alone, it was able to enhance the number of cell cycle withdrawals when given to cells immediately following release from CDK4/6 inhibition. A number of genotoxic anti‐cancer drugs also induce replication stress; therefore, we analysed the effect of three such compounds that impede DNA replication differently: camptothecin (TopoI inhibitor), doxorubicin (TopoII inhibitor) or olaparib (PARP inhibitor). We chose a dose of each drug previously shown to be sublethal in RPE1 cells (Olivieri *et al*, 2020), and demonstrated that this produced only mild effects on cell cycle timing and progression in control cells (Fig 5C–E). However, when given following a palbociclib arrest, these drugs caused the majority of cells to fail to complete the first cell cycle. In particular, there was a large increase in cells that commenced DNA replication but then withdrew into G1 before entering mitosis. Colony forming assays demonstrated an increased sensitivity to the drug combinations compared with the monotherapies, with doxorubicin and olaparib causing a strong reduction in proliferation after only 1 day of palbociclib treatment (Fig 5F and G). These data suggest that consecutive treatment with CDK4/6 inhibitors and existing genotoxic drugs may be a promising therapeutic strategy, as also demonstrated recently by others, but for different reasons (Roberts *et al*, 2020) (see Discussion).

**Figure EV4:**
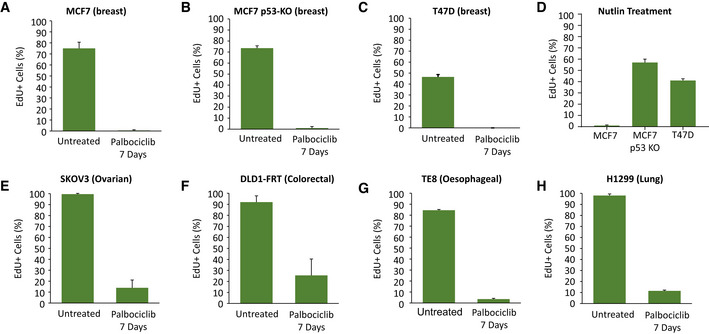
EdU staining to quantify cell cycle arrest in different tumour lines treated with palbociclib or nutlin A–HQuantification of the percentage of cells undergoing S‐phase during the final 24 h of a 7‐day arrest with 1 µM palbociclib in the tumour lines indicated (A–C and E–H), or the final 24 h of a 3‐day arrest with 5 µM Nutlin in the breast cancer lines indicated (D). Cells were treated with DMSO (untreated) or palbociclib (1 μM) for 7 days, or Nutlin for 3 days, with EdU (10 μM) pulsed in for the last 24 h of treatment. Data show mean + SD from two experiments, with at least 100 cells quantified per experiment.

**Figure 6:**
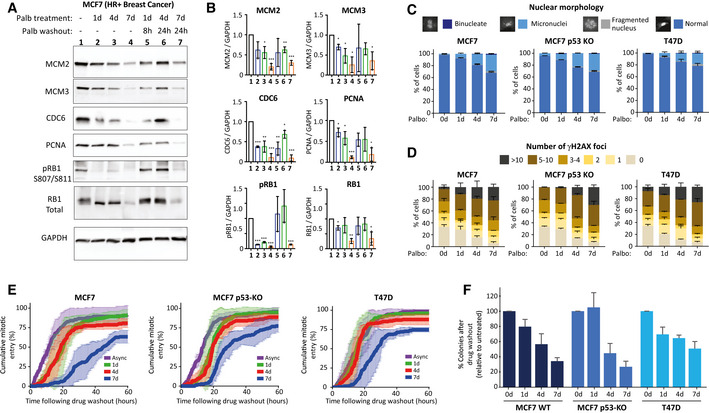
Prolonged G1 arrest following palbociclib treatment in breast cancer cells downregulates replisome components, causes DNA damage and reduces long‐term proliferation ARepresentative western blots of whole‐cell lysates from MCF7 cells treated with palbociclib (1 μM) for 1, 4 or 7 days, or treated identically, and then washed out for the indicated times to allow cells to enter S‐phase.BAnalysis of adjusted relative density from three independent western blot experiments. Bars display mean values ± SD. Significance determined by unpaired Student's *t*‐test comparing treated target protein to asynchronous target control (*< 0.01, **< 0.001, ***< 0.0001).C, DQuantification of the nuclear morphologies (C) or γH2AX‐positive DNA damage foci (D) from MCF7, MCF7 p53 KO or T47D cells that were treated with palbociclib (1 μM) for 0, 1, 4 or 7 days and then analysed 72 h after drug washout. Either 100 cells (nuclear morphology) or 50 cells (γH2AX) were scored per condition per experiment and bar graphs represent mean data + SEM from three experiments.ECumulative mitotic entry of cells following washout from 0, 1, 4 or 7 days treated of palbociclib (1 μM). A total of 50 cells were quantified per experiments and graphs display mean ± SEM from three experiments.FQuantification of colony forming assays of MCF7, MCF7 p53 KO and T47D cells treated with CDK4/6 inhibitor for 0, 1, 4 or 7 days and then grown at a low density without palbociclib for 14–21 days. Bar graphs display mean data + SEM from 4 to 5 experiments.

We next sought to examine whether these results were broadly applicable to tumour types that are known to be responsive to CDK4/6 inhibitor treatment. We first chose to test a well‐established p53‐positive HR^+^ breast cancer line, MCF7, which we verified could hold a prolonged arrest for up to 7 days following 1 µM palbociclib treatment (Fig EV4A). Importantly, during this arrest, various replisome components were downregulated, and these also remained low following drug washout (Fig 6A and B), in a manner that was comparable to the effects seen in RPE1 cells (Fig 4D and E). To examine if this induced replication stress and a p53‐dependent cell cycle arrest following drug washout, we generated p53‐KO MCF7s by CRISPR/Cas9 gene editing (Appendix Fig S5) and compared these to a well‐established p53‐null HR^+^ breast tumour line, T47D, both of which could also arrest efficiently in 1 µM palbociclib for up to 7 days (Fig EV4B and C). All three breast cancer lines displayed an increase in micronuclei and γH2AX foci after release from prolonged palbociclib arrest (Fig 6C and D), which implies that replication stress is induced following drug washout, as in RPE1 cells (Fig 4). However, a notable difference was the fact that p53‐WT MCF7 cells had elevated levels of DNA damage compared to RPE1‐WT cells, both basally and following drug washout. They also formed micronuclei more readily after drug washout, which suggested that mitotic entry was not prevented to the same extent in p53‐proficient MCF7s. We confirmed that p53 could indeed induce a cell cycle arrest by culturing cells in 5 µM nutlin, which efficiently arrested p53‐WT MCF7 cells, but not p53‐KO MCF7s or T47D (Fig EV4E). To assess this further, we performed time‐lapse imaging to quantify mitotic entries over time following CDK4/6 inhibitor washout. This demonstrated that prolonged CDK4/6 inhibition in breast cancer lines prevented and delayed mitotic entry (Fig 6E), as expected if cell cycle progression was defective. However, the effect of p53 loss appeared to be modest, which potentially explains why DNA damage foci are not dramatically different between p53‐proficient and p53‐null breast cancer lines, in comparison to RPE1 cells (compare Figs 3D and E with 6C and D). This is also consistent with colony forming assays which showed that long‐term palbociclib treatment reduces proliferative potential in breast cancer lines, but this inhibition was less pronounced than in RPE1 cells, and it was not markedly affected by p53 status (Fig 6F). Our interpretation of these data is that while p53 is an important barrier to proliferation in cells with replication stress and DNA damage, cancer lines with functional p53 either exhibit an impaired p53 response, or alternatively, have adapted in other ways to maintain proliferative capacity in the presence of genomic damage (see Discussion). In summary, long‐term palbociclib treatment induces DNA damage in breast cancer cells, which is likely caused by the downregulation of replisome components during the G1 arrest and replication stress following drug release. This stress is able to restrain long‐term proliferation, but not as effectively as observed in RPE1 cells, perhaps because cancer lines have evolved to proliferate in the presence of genotoxic stress.

**Figure 7:**
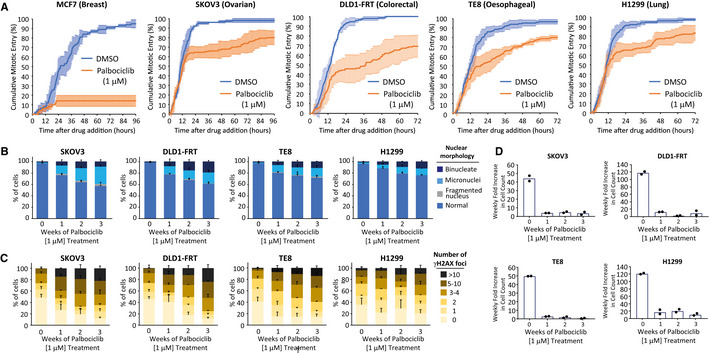
Constitutive palbociclib treatment in various tumour lines causes cell cycle delays and DNA damage AQuantification of cumulative mitotic entry over time immediately following 1 μM palbociclib treatment in a range of tumour cell lines, as indicated. Mitotic entry of 50 cells per condition per experiment were analysed immediately following drug addition. Graph shows cumulative mean ± SD from three experiments (150 cells total).B, CQuantification of the nuclear morphologies (B) or γH2AX‐positive DNA damage foci (C) from different cancer lines, as indicated, treated continuously with palbociclib (1 μM) for 0, 1, 2 or 3 weeks. Either 100 cells (nuclear morphology) or 50 cells (γH2AX) were scored per condition per experiment and bar graphs represent mean data + SEM from three experiments.DQuantification of weekly proliferation rate in cells treated as in (B). A total of 60,000 cells of each cell line were cultured in 10 cm dishes with 1 μM palbociclib for a total of 3 weeks. Every 7 days, cells were trypsinized and total cell counts were determined. The 0‐week time point shows the 7‐day fold increase of untreated cells. The data points show fold increase in cell count over each 7‐day period, and bars represent the mean from two experiments.

To examine if replication stress is also induced in other cancer types following CDK4/6 inhibition, we screened a panel of p53‐null tumour lines for their ability to arrest in 1 µM palbociclib. While proliferation was significantly slowed in a range of cancer types, we were unable to find tumour cells that could fully arrest for 7 days in 1 μM palbociclib, as determined by EdU incorporation after 6 days of the arrest (Fig EV4E–H). Time‐lapse imaging to quantify mitotic entries after drug addition showed that this proliferative arrest is associated with cell cycle delays that occurred prior to S‐phase because cells that had passed the restriction point entered mitosis at a normal rate for approximately the first 12 h of the movie (Fig 7A). Similar delays have recently been shown to occur in G1 upon partial CDK4/6 inhibition (Tan *et al*, 2021), an effect that we also see in RPE1‐FUCCI cells when the dose of palbociclib is decreased below 1 μM, allowing G1 to be extended for up to 3 days (Appendix Fig S6). We hypothesized that G1 delays could be problematic for cancer lines that are already subject to endogenous replication stress, therefore we quantified nuclear morphology and DNA damage in cells treated continuously with 1 µM palbociclib for up to 3 weeks. Figure 7B and C shows that micronuclei and γH2AX foci are progressively induced over time in these palbociclib‐treated cancer cells, implying that replication stress can be induced in various cancer lines even when proliferation cannot be fully blocked by palbociclib treatment (see Appendix Fig S7 for representative images). This DNA damage is associated with a strong reduction in proliferative potential throughout the course of palbociclib treatment (Fig 7D). Therefore, CDK4/6 inhibition can cause genotoxic stress in a wide variety of tumour types, and this can occur during periods of continual drug treatment, as long as cell cycle progression is not completely inhibited during that time.

## Discussion

A major finding of this study is that CDK4/6 inhibitors, like many other broad‐spectrum anti‐cancer drugs, induce genotoxic stress during S‐phase. This may initially appear counterintuitive for a class of drugs that effectively prevent S‐phase entry. However, when cells are arrested in G1 following CDK4/6 inhibition, key components of the replisome are downregulated and, if this is allowed to proceed for too long, then DNA replication is perturbed upon release from that G1 arrest. Therefore, we propose that long‐term cell cycle withdrawal following CDK4/6 inhibition depends on at least two key factors: (i) the duration of time that cells remain arrested in G1, and (ii) how well cells can then tolerate the resulting replication stress.

Using a non‐transformed near‐diploid RPE1 cell line, we demonstrate that the G1 arrest becomes problematic if it lasts for longer than 2 days. We predict that this is the combined result of poor origin licencing due to downregulation of the MCM complex, and reduced levels of replication fork proteins that are needed after those origins fire in S‐phase. Cells are able to tolerate mild reductions in MCM proteins due to the presence of excess dormant replication origins, but if these levels fall too much, then cells become hypersensitive to replication stress (Ge *et al*, 2007; Ibarra *et al*, 2008). This probably explains why 1 day of palbocicilib treatment is tolerable in RPE1 cells, despite a reduction in licenced origins, and why this is no longer tolerable when replication stress is elevated pharmacologically (Figs 4F and 5F and G). It is likely that tumour cells that are subject to constitutive replication stress (Macheret & Halazonetis, 2015) will be similarly sensitive to short durations of G1 arrest. In agreement with this hypothesis, breast cancer lines start to exhibit DNA damage and micronuclei after as little as 1 day of palbociclib arrest (Fig 6C and D), and other cancer lines experience significant DNA damage by palbociclib treatment that can only moderately slow cell cycle progression (Fig 7A–C). To better understand how cell lines respond differently to CDK4/6 inhibition in the future, it will be important to identify why DNA replication factors are downregulated during a G1 arrest and to compare if the level of this downregulation varies between different cell types. This may ultimately help to reveal sensitive genotypes or biomarkers that can predict response.

Our comparisons between RPE1 and breast cancer cells did reveal some important differences in how these cells respond to CDK4/6 inhibition. Although both cell types experienced DNA damage following palbocicilib treatment, the ability of this DNA damage to restrain cell cycle progression was reduced in the breast cancer lines compared to RPE1 cells: Specifically, 7 days of palbociclib treatment was needed in MCF7s to reduce long‐term proliferation to a similar extent as is observed after 3 days of treatment in RPE1 cells (compare Figs 5G and 6F). This could be due to a weakened p53 response in MCF7 cells, given that MDM2 is amplified/overexpressed and the MDM2 inhibitor p14ARF is lost due to deletion of the CDKN2A/ARF locus (Gudas *et al*, 1995; Stott *et al*, 1998; Maglic *et al*, 2013). Alternatively, or in addition, the type or extent of DNA damage induced in RPE1 cells maybe more effective at activating the p53 pathway. In relation to this, it is notable that breast cancer cells rarely produced fragmented nuclei that are indicative of catastrophic chromosome segregation errors during mitosis, but instead primarily produced micronuclei that form when one or two chromosomes pairs fail to segregate correctly (Figs 3D and 6C); an event that does not commonly lead to p53 activation (Santaguida *et al*, 2017). Interesting in this regard is also the observation that some replisome components such as PCNA and MCM2 are downregulated more extensively in RPE1 cells in comparison to MCF7s (compare Figs 4D and E with 6A and B).

It is important to highlight that the RPE1 cells used in this study, although telomerase immortalized and non‐transformed (Bodnar *et al*, 1998), do still have mutations in at least two known cancer‐associated genes: *CDKN2A* and *KRAS* (Di Nicolantonio *et al*, 2008; Libouban *et al*, 2017). *CDKN2A* deletion causes sensitivity to palbociclib (Ramsey *et al*, 2007; Dean *et al*, 2010; Wiedemeyer *et al*, 2010; Young *et al*, 2014), therefore the CDKN2A mutation may explain why RPE1 cells arrest so efficiently in G1 following CDK4/6 inhibition (the IC50s for palbociclib and abemaciclib are comparable to the most sensitive cancer cell type in a panel of 560 tested lines: see Fig 1A and (Gong *et al*, 2017)). It is also possible that the activating *KRAS* mutation (Di Nicolantonio *et al*, 2008) contributes to the phenotype of CDK4/6 inhibition; for example, by causing oncogene‐induced replication stress (Kotsantis *et al*, 2018).

There is an urgent need to identify biomarkers that can predict long‐term response to CDK4/6 inhibitors (Migliaccio *et al*, 2021), and we hypothesize that candidates in this regard will fall into at least three categories: (i) factors that determine the initial response in G1, for example, by controlling the efficiency of the G1 arrest or the extent that replisome components are downregulated; (ii) factors that control how well the resulting stress during S‐phase is tolerated, for example, mutants that induce replication stress or inhibit the ability of cells to repair replication defects; and (iii) downstream mediators that determine the fate of cells following this genotoxic stress. Our data demonstrate that p53 is a critical downstream fate determinant that controls cell cycle withdrawal in RPE1 cells, and this may help to explain why p53 loss has been associated with resistance to CDK4/6 inhibition in the clinic (Patnaik *et al*, 2016; Wander *et al*, 2020). It should be noted, however, that specific knockout of p53 in MCF7 cells did not dramatically alter long‐term cell cycle progression or DNA damage following release from long‐term CDK4/6 inhibition. Therefore, whether p53 can cause cell cycle withdrawal after CDK4/6 inhibition is probably cell line dependent. As discussed above, p53‐proficient cells could still have a weakened p53 response due to alterations in MDM2/ARF, and the status of other pathways that allow the cell to respond to replication stress, such as ATR, are probably also crucial. It will be important in future to carefully address the role of p53 and other DNA damage response pathways in a wide variety of cancer lines, by focusing specifically on how these pathways define the response to a prolonged G1 arrest.

In clinical practice, palbociclib and ribociclib are given in cycles of 3 weeks on and 1 week off; primarily to allow recovery from haematopoietic toxicity (Pernas *et al*, 2018). It is possible that these drug‐holiday periods contribute to tumour cell killing by allowing replication stress to cause DNA damage. It is important to test this hypothesis because, if elevated DNA damage is detected when CDK4/6 inhibitors are withdrawn, then the timing/duration of drug holidays could potentially be optimized. However, in addition to this, continual CDK4/6 inhibitor dosing could also induce genotoxic damage if this dose causes cell cycle delays instead of a complete G1 arrest, as we observed in a range of tumour types that cannot be fully arrested by palbociclib (Fig 7). We predict this damage is caused by extended G1 lengths that are induced by partial CDK4/6 inhibition (Tan *et al*, 2021) (Appendix Fig S6) and/or reductions in E2F targets that are required for S‐phase. Therefore, replication stress may be ongoing in patients during the periods of CDK4/6 inhibitor treatment, assuming that not all tumour cells will be held fully in G1 throughout this treatment period. It will be important in the future to measure replication stress directly in breast cancer patients treated with CDK4/6 inhibitors, and to compare this in patients who are dosed continuously, as with abemaciclib, or in repeated on/off cycles, as with palbociclib/ribociclib (Pernas *et al*, 2018).

An important finding of this work is that CDK4/6 inhibition sensitizes RPE1 cells to cytotoxic chemotherapeutics currently in widespread clinical use. This was also demonstrated recently in pancreatic ductal adenocarcinoma (PDAC); a tumour type that is similarly characterized by activating *KRAS* (G12V) and *CDKN2A* mutations (Salvador‐Barbero *et al*, 2020). In this case, the sensitivity in PDAC models was attributed to the ability of palbociclib to prevent DNA repair. It is possible that CDK4/6 inhibition promotes genotoxic damage by both elevating replication stress *and* inhibiting DNA repair, however, it is important to carefully distinguish between these possibilities because it may determine the optimal order of drug exposure. In the Salvador‐Barbero *et al* study, CDK4/6 is inhibited *after* genotoxic damage to reduce DNA repair (Salvador‐Barbero *et al*, 2020), whereas in this study, CDK4/6 is inhibited *prior* to genotoxic damage to elevate replication stress. The promises and dangers surrounding the combined use of CDK4/6 inhibitors and cytotoxic chemotherapeutics are discussed at length in this review (Roberts *et al*, 2020), which contains details of the different tumour types that have been shown to benefit from this approach. Particularly interesting in this regard are phase II trial results in triple‐negative breast cancer (TNBC) patients showing that combining the CDK4/6 inhibitor trilaciclib with the genotoxic agents gemcitabine and carboplatin improves overall survival (Tan *et al*, 2019). This study was designed to protect myeloid lineages from chemotherapeutic toxicity; therefore, this enhanced response may be due to the preservation of cytotoxic T cells during chemotherapy. Considering that transient CDK4/6 inhibition can also activate this T‐cell population (Goel *et al*, 2017; Chaikovsky & Sage, 2018; Deng *et al*, 2018; Schaer *et al*, 2018), these effects could combine to enhance immune‐mediated tumour clearance. However, it is also possible that the benefit reflects enhanced genotoxic damage within tumour cells, if trilaciclib is able to slow proliferation and cause replication stress in these cells. This may explain why overall survival was not enhanced by similar trilaciclib/genotoxic combinations in small cell lung cancer (Weiss *et al*, 2019; Daniel *et al*, 2020; Hart *et al*, 2021), which are almost always Rb null and therefore insensitive to CDK4/6 inhibitors (George *et al*, 2015) (in comparison, Rb inactivation is only observed in 7–20% of TNBCs (Johnson *et al*, 2016; Peck *et al*, 2021; Rocca *et al*, 2017)). TNBC subtypes that arrest following CDK4/6 inhibition have recently been identified (Asghar *et al*, 2017; Peck *et al*, 2021), therefore the ability of CDK4/6 inhibitors to enhance the effect of cytotoxic chemotherapeutics in these subtypes warrants investigation.

Finally, CDK4/6 inhibition can re‐sensitize tumours to immune checkpoint blockade (Xue *et al*, 2007; Krizhanovsky *et al*, 2008; Kang *et al*, 2011; Acosta *et al*, 2013; Goel *et al*, 2017; Chaikovsky & Sage, 2018; Deng *et al*, 2018; Jerby‐Arnon *et al*, 2018; Ruscetti *et al*, 2018, 2020). The ability of CDK4/6 inhibition to inhibit DNA replication could help to promote immune engagement in a number of different ways. Replication stress can activate the cGAS/STING‐mediated interferon (IFN) response and increase the number of mutations/neoantigens (Ubhi & Brown, 2019; Ragu *et al*, 2020). In addition, it can induce senescence, thereby creating non‐proliferative tumour cells that continually secrete factors to engage the immune system (Xue *et al*, 2007; Krizhanovsky *et al*, 2008; Kang *et al*, 2011; Acosta *et al*, 2013). CDK4/6 inhibitor‐induced senescence helps to sensitize tumours to immune checkpoint blockade (Jerby‐Arnon *et al*, 2018; Ruscetti *et al*, 2018, 2020), therefore it will be important to test whether replication stress leads to senescence in these settings. There are currently 14 clinical trials ongoing in 8 tumour types to assess whether CDK4/6 inhibition can improve response to anti‐cancer immunotherapy (Wagner & Gil, 2020), therefore it will also be important to assess whether p53 status correlates with response in these situations. p53 inactivation could potentially enhance immune engagement by allowing severely under‐replicated chromosomes to progress into mitosis and produce micronuclei that can activate the cGAS‐STING pathway (Bartsch *et al*, 2017; Gekara, 2017; Harding *et al*, 2017; Mackenzie *et al*, 2017). However, it could also block senescence entry (Mijit *et al*, 2020) and enhance heterogeneity, thereby increasing the chances of immune evasion.

In summary, the work presented here links CDK4/6 inhibitors with genotoxic stress, which now provides a rationale to better understand how these drugs selectively target tumour cells. CDK4/6 inhibitors are already known to arrest tumour cells more efficiently in G1 (Gong *et al*, 2017; Alvarez‐Fernandez & Malumbres, 2020), but if they also capitalize on the fact that these tumours are also exquisitely sensitive to that arrest as a result of ongoing replication stress, then the implications for cancer treatment could be wide ranging. It is therefore now critical to build on this work and carefully examine these concepts in preclinical and clinical settings to determine whether replication stress is a common outcome of CDK4/6 inhibition.

## Materials and Methods

### Cell culture and reagents

hTERT‐RPE1 (RPE1) was purchased from ATCC and the RPE1‐FUCCI was published previously (Krenning *et al*, 2014). The human ER^+^ breast cancer lines, MCF7 and T47D, were purchased from ATCC. The human oesophageal squamous carcinoma line, TE8, was obtained from the Cell Resource Centre for Biomedical Research, Institute of Development, Aging and Cancer, Tohoka University, Japan. The human ovarian adenocarcinoma line, SKOV3, was acquired from CRUK. The human non–small cell lung carcinoma line, H1299, was purchased from ATCC. The human colorectal adenocarcinoma line, DLD1‐FRT, was a kind gift from Stephen Taylor, which has been published previously (Girdler *et al*, 2006). All cells were authenticated by STR profiling (Eurofins) and screened for mycoplasma every 1–2 months. All cells were cultured at 37°C with 5% CO_2_ in DMEM (Thermo Fisher Scientific, Gibco 41966029) supplemented with 9% FBS (Thermo Fisher Scientific, Gibco 10270106) and 50 μg/ml penicillin/streptomycin, except H1299 and TE8 which were cultured in RPMI (Thermo Fisher Scientific, Gibco 21875034) supplemented with 9% FBS and 50 μg/ml penicillin/streptomycin. The following drugs were used in this study: Palbociclib (PD‐0332991, hydrochloride salt, MedChemExpress, HY‐50767A); ribociclib (LEE‐011, Selleckchem, S7440); abemaciclib (LY‐2835219, Selleckchem, S7158); trilaciclib (G1T28, Insight, HY‐101467A); S‐Trityl‐L‐cysteine (STLC; Sigma Aldrich, #164739), aphidicolin (Santa Cruz, SC‐201535), doxorubicin (Selleckchem, S1208), olaparib (Selleckchem, S1060), camptothecin (Sigma, C9911), nutlin‐3a (Sigma, SML0580), DAPI (4′,6‐Diamidino‐2‐Phenylindole; Thermo Fisher Scientific, D1306), RO‐3306 (Tocris, #4181), EdU (Sigma‐Aldrich, BCK‐EDU488), nocodazole (Sigma‐Aldrich, #487928) and VE‐821 (Selleckchem, S8007).

### Cell density

To prevent cell–cell contact from inhibiting exit from G1, it was crucial to plate cells at low density for all experiments, but especially for RPE1 cells that exit the cell cycle upon contact inhibition. Therefore, cells were plated at a maximum density of 8,000 cells per cm^2^ immediately prior to the arrest with CDK4/6 inhibitors.

### Immunofluorescence

Cells were plated at low density on High Precision 1.5H 12‐mm coverslips (Marienfeld) and fixed for 10 min with 4% paraformaldehyde dissolved in PBS. Once fixed, coverslips were washed three times in PBS and then blocked in 3% BSA dissolved in PBS with 0.5% Triton X‐100 for 30 min. Coverslips were then incubated with primary antibodies at 4°C overnight, prior to washing with PBS and incubation with secondary antibodies and DAPI (1 μg/ml) for 2–4 h at room temperature. After further washing, coverslips were mounted onto slides with ProLong Gold Antifade (Thermo Fisher Scientific, P10144). Coverslips were imaged on either a Zeiss Axio Observer using a Plan‐apochromat 20×/0.8 M27 Air objective or a Deltavision with a 100×/1.40 NA U Plan S Apochromat objective. The primary antibodies used were as follows: rabbit anti‐p21 (H‐164; Santa Cruz, sc‐756; 1/500), mouse anti‐p53 (clone DO‐1; Santa Cruz, sc‐126; 1/1,000), mouse anti‐phospho‐Histone H2A.X (Ser139; clone JBW301; Sigma, 05‐636; 1/1,000) and rabbit anti‐53BP1 (Novus biologicals, NB100‐304; 1/1,000). The secondary antibodies used were highly cross‐absorbed goat anti‐rabbit or anti‐mouse coupled to Alexa Fluor 488 and Alexa Fluor 568 which were all used at 1/1,000 dilution. All antibodies were made up in 3% BSA in PBS. For EdU staining, a base click EdU staining kit was used (Sigma, BCK‐EDU488), as per manufacturer's instructions.

### MiDAS protocol

RPE1 p53 KO cells were plated at low confluence in 10 cm dishes and treated with palbociclib (1.25 μM) for 7 days. Palbociclib was then removed via an extensive washout and cells were transferred coverslips. Coverslips were then returned to incubation for 16 h before being treated with RO‐3306 (10 µM) for a further 2 h to enrich for cells in G2. Media were then exchanged twice over 15 min and cells were treated with EdU (10 µM) and nocodazole (3.3 µM). After 1 h, cells were fixed in 4% PFA. Note that control cells were either treated with aphidicolin (0.4 µM) for 40 h or left untreated prior to addition of RO‐3306. Following fixation cells were permeabilized with 0.2% Triton X‐100 in 3% BSA dissolved in PBS. Staining of incorporated EdU was carried out as per manufacturers' instructions and coverslips were mounted onto slides using prolong gold antifade. Coverslips were imaged using a Deltavision with a 100×/1.40 NA U Plan S Apochromat objective.

### Time‐lapse imaging

For FUCCI time‐lapse imaging, cells were plated at low density (approximately 15,000 cells per well) and imaged in 24‐well plates in DMEM inside a heated 37°C chamber with 5% CO_2_. Images were taken every 10 min with a 10×/0.5 NA air objective using a Zeiss Axio Observer 7 with a CMOS ORCA flash 4.0 camera at 4 × 4 binning. GFP‐53BP1/H2B‐RFP RPE1 cell lines were imaged on a DeltaVision Elite system in eight‐well chambers (Ibidi) in L15 media within a heated 37°C chamber. Images were taken every 4 min with a 40×/1.3 NA oil objective using a DV Elite system equipped with Photometrics CascadeII: 1024 EMCCD camera at 4 × 4 binning. For bright‐field imaging, cells were imaged in a 24‐well plate in DMEM in a heated chamber (37°C and 5% CO_2_) with a 10×/0.5 NA air objective using a Hamamatsu ORCA‐ER camera at 2 × 2 binning on a Zeiss Axiovert 200 M, controlled by Micro‐manager software (open source; https://micro‐manager.org/) or with a 20×/0.4 NA air objective using a Zeiss Axio Observer 7 (details above). For the time lapse shown in Fig 7A, cells were plated into Essen Imagelock 96‐well plates and imaged using a Sartorius IncuCyte ZOOM using a 10× objective and 15‐min imaging intervals.

### Generating knockout cell lines

To generate p53 knockout cells, a gRNA targeting exon 4 of p53 (ACCAGCAGCTCCTACACCGG) was cloned into the pEs‐gRNA vector by site‐directed mutagenesis, as described previously (Munoz *et al*, 2014). RPE1 and RPE1‐FUCCI cells were then transfected with this gRNA vector along with a pcDNA5‐Cas9 vector in a 3:1 ratio with Fugene HD. MCF7 and RPE1‐53BP1‐H2B cells were transfected with same ratio but using Lipofectamine™ 2000 Transfection Reagent (Thermo Fisher Scientific). Knockout cells were subsequently selected by cultured in 5 μM of Nutlin‐3a until no visible cells remained on the control non‐transfected plates (approximately 3 weeks). p53 knockout status was confirmed via immunoblotting and immunofluorescence.

### Western blotting

Total protein lysates for immunoblot were prepared by harvesting cells in trypsin, pelleting and flash freezing. Cell pellets were lysed in ice‐cold RIPA buffer (50 mM Tris ph 7.6, 150 mM NaCl, 1% NP40, 0.5% sodium deoxycholate, 1 mM EDTA, 0.1% SDS and protease and phosphatase inhibitors [0.1 mM Pefabloc, 1 μg/ml pepstatin A, 1 μg/ml leupeptin, 1 μg/ml aprotinin, 10 μg/ml phosvitin, 1 mM β‐glycerol phosphate and 1 mM sodium orthovanadate]) on ice for 20 min. Lysates were centrifuged at 13,000 *g* and 4°C for 10 min, followed by Bradford assay (Biorad) to determine equal amounts of protein to load per lane. Samples were mixed with loading buffer to final concentrations of: 1% SDS, 2.5% 2‐mercaptoethanol, 0.1% bromophenol blue, 50 mM Tris, pH 6.8 and 10% glycerol. Samples were boiled, then separated on SDS—PAGE gels and transferred polyvinylidene difluoride membranes (Thermo Fisher Scientific). After transfer, blots were blocked in 5% milk in TBS with 0.1% Tween 20 (TBS‐T) and incubated overnight at 4°C in primary antibody in TBS‐T. Then, membranes were washed in TBS‐T 3x, incubated in HRP‐conjugated secondary antibody for 1 h at RT, washed in TBST 3x and imaged with ECL Prime (Amersham). Membranes were stained with Ponceau S (Sigma‐Aldrich) to visualize protein loading. Antibodies used were mouse anti‐MCM2 (BM28; Clone 46; BD Biosciences, 610701; 1/1,000), rabbit anti‐MCM3 (A300‐192A, Bethyl Laboratories; 1/1,000), mouse anti‐CDC6 (Clone 180.2; sc‐9964, Santa Cruz; 1/500), mouse anti‐PCNA ((lone F‐2; sc‐25280, Santa Cruz; 1/1,000), mouse anti‐RB (554136, BD Biosciences), rabbit anti‐pRB (Clone G3‐245; Ser807/Ser811; 9308S, Cell Signaling Technology; 1/1,000), rabbit anti‐p21 (H‐164; Santa Cruz, sc‐756; 1/250), mouse anti‐p53 (Clone DO‐1; Santa Cruz, sc‐126; 1/1,000) and rabbit anti‐actin (Sigma, A2066; 1/5,000). Anti‐Mouse HRP (1858413, Pierce; 1/10,000) and anti‐Rabbit HRP (31460, Pierce; 1/10,000) secondary were used.

### Chromatin‐bound MCM FACS assays

The amount of DNA‐loaded MCM following release from palbociclib treatment was analysed as described previously (Matson *et al*, 2017). RPE1 WT or p53 KO cells were treated with palbociclib for 1 or 7 days and the drug was washed out for 8 or 24 h respectively. Thirty minutes prior to cell collection, cells were pulse labelled with 10 μM EdU (Sigma) to monitor DNA synthesis. Soluble MCM was pre‐extracted from cells on ice for 10 min in cold CSK buffer (10 mM Pipes pH 7.0, 300 mM sucrose, 100 mM NaCl and 3 mM MgCl_2_) supplemented with 0.5% triton X‐100, protease inhibitors (0.1 mM AEBSF, 1 μg/ml pepstatin A, 1 μg/ml aprotinin and 0.1 mM PMSF) and phosphatase inhibitors (10 μg/ml phosvitin, 1 mM β‐glycerol phosphate and 1 mM Na‐orthovanadate). After washing cells in PBS + 1% BSA, cells were fixed in PBS + 4% paraformaldehyde (Sigma) for 15 min at room temperature. Cells were processed for EdU conjugation to Alexa Fluor 647‐azide (Life Technologies) by incubation in PBS containing 1 mM CuSO_4_, 1 mM AF‐647 and 100 mM fresh ascorbic acid for 30 min at room temperature in the dark. The levels of DNA‐loaded MCM were detected by incubating cells in anti‐MCM2 BM28 antibody (1:200, BD biosciences, 610700) for 1 h at 37°C in the dark followed by incubation in anti‐mouse 488 secondary antibody (1:1,000) for 1 h at 37°C in the dark. DNA content was measured by incubating cells in 1 μg/ml DAPI and 100 μg/ml RNAase for 1 h at 37°C in the dark or alternatively overnight at 4°C. Cells were analysed using an Attune NxT flow cytometer (Thermo Fisher Scientific) and data were analysed using FCS Express 7 Research (De Novo Software).

For each experimental condition, an identically treated sample was included but was not labelled for EdU or MCM in order to determine the limit of detection. Early S‐phase cells were analysed by gating on cells that had 2C DNA content and were EdU positive. For each sample, the mean AF‐488 fluorescent intensity of early S‐phase cells was divided by the mean AF‐488 fluorescent intensity of the identically treated but unstained control sample. The displayed data are the normalization of these ratios to asynchronous control cells.

### Colony‐forming assay

For the colony‐forming assays, cells were treated with palbociclib (1.25 µM), ribociclib (5 µM), abemaciclib (600 nM) or trilaciclib (600 nM) at 200,000 cells per 15 cm dish for different length of time (1–7 days) prior to drug washout (6 × 1 h washes). Following washing and trypsinization, RPE1s were plated in triplicate at 250 cells into 10 cm dishes and left to grow for 10 days, whereas MCF7 and T47Ds were plated at 250 cells in triplicate in 6‐well plates and allowed to grow for 14–21 days. For the experiments in Fig 5, different genotoxic drugs were added for the first 24 h after replating, before washout and incubation in standard media for the remaining 9 days. At the end of the assay, cells were washed twice in PBS and then fixed at 100% ethanol for 5 min. Developing solution (1:1 ratio of 2% Borax:2% Toluene‐D in water) was added to the fixed cells for 5 min and the plates were then rinsed thoroughly with water and left to dry overnight. The plates were then scanned and the number of colonies were quantified using ImageJ. This was performed by cropping to an individual plate and converting to a binary image. The fill holes, watershed and analyse particles functions were then used to count colonies.

### Weekly fold increase in cell count

A total of 60,000 cells from each line were plated into 10 cm dishes and treated with palbociclib (1 µM) or DMSO (control). After 7 days of treatment, cells were trypsinized, total cell counts were determined and the 7‐day fold increase in cell count was calculated. From the cell suspension, 60,000 cells were returned to palbociclib treatment, and this process was repeated two more times for a total of 3 weeks. At each time point, excess cells were transferred to coverslips and taken for immunofluorescence with γH2AX antibodies.

### Mass spec sample preparation

Cells were plated in 15 cm dishes and treated with palbociclib for 2 or 7 days. Cells were lysed in cell extraction buffer containing 2% SDS, 1X PhosStop (Roche) and 1× cOmplete protease inhibitor cocktail (Roche). An aliquot of extract containing 100 µg protein was then digested by benzonase (Merck) and precipitated by acetone. The protein pellet was resuspended in digest buffer (0.1 M triethylammonium bicarbonate, pH 8.5, Sigma‐Aldrich, tandem mass tag [TMT] labelling using a six‐plex TMT kit [Thermo Fisher Scientific] and desalted. Peptides were then separated using high pH reverse phase chromatography (Waters BEH 4.6 mm × 150 mm C18 column; A, 10 mM ammonium formate, pH 9.0; B, 80% acetonitrile plus 10 mM ammonium formate, pH 9.0) into 16 fractions (Hiraga *et al*, 2017). Fractions were then dried under vacuum and resuspended in 5% formic acid for liquid chromatography tandem mass spectrometry (LC‐MS/MS) analysis.

### LC‐MS/MS

LC‐MS analysis was performed on an Orbitrap Fusion Lumos Tribrid MS (Thermo Fisher Scientific) coupled online, to an Ultimate 3000 RSLCnano HPLC (Dionex, Thermo Fisher Scientific). Peptides were separated on a 50 cm EASY‐Spray column (Thermo Fisher Scientific) and ionized using an EASY‐Spray source (Thermo Fisher Scientific) operated at a constant temperature of 50°C. Mobile phase A consisted of 0.1% formic acid in water while mobile phase B consisted of 80% acetonitrile and 0.1% formic acid. Peptides were loaded onto the column at a flow rate of 0.3 μl/min and eluted at a flow rate of 0.25 μl/min according to the following gradient: 2–40% mobile phase B in 120 min, then to 95% in 11 min. The percentage of mobile phase B remained constant for 10 min and returned to 2% until the end of the run (160 min).

MS1 survey scans were performed at 120,000 resolution (scan range 350–1,500 *m*/z) with an ion target of 2.0 × 10^5^ and maximum injection time of 50 ms. For MS2, precursors selected using a quadrupole isolation window of 1.2 Th with an AGC target of 1E5 and a maximum injection time of 100 ms. Product ions from HCD fragmentation (32% normalized collision energy) were then scanned using the Orbitrap with 30k resolution). Only ions with charge between 2 and 7 were selected for MS2.

### MS data analysis

Raw data files were processed using MaxQuant version 1.6.2.6 (Cox & Mann, 2008), which incorporates the Andromeda search engine (Cox *et al*, 2011). The spectra were searched against a human FASTA database (accessed June 2018) containing all reviewed entries in the reference UniProt Human Proteome. The processed output was then analysed using R or RStudio software. In replicate three of the MS analysis, almost all protein intensities in the 7‐day palbociclib treatment group were zero, indicating improper TMT labelling. Therefore, this experimental group was excluded from further analysis.

### Image quantification

To calculate the percentage of G1‐arrested cells (in Figs 1A and B, 2A and EV1), RPE1‐FUCCI cells were treated (as described in the legends) and then imaged using a Zeiss Axio Observer 7 with 10×/0.5 NA air objective and a CMOS ORCA flash 4.0 camera at 4 × 4 binning. Five positions were imaged per well using filtersets to image mKO2‐cdt1 (red) and mAG‐geminin (green). The TrackMate function in ImageJ was then used to quantify the number of RPE1‐FUCCI cells in each channel. The percentage of red (G1 arrested) cells was calculated and used to generate dose–response curves in GraphPad Prism 7.

The single‐cell FUCCI profiles were generated manually by analysing RPE1‐FUCCI movies. A total of 50 red cells were randomly selected and marked at the beginning of the movie. The time points in which the FUCCI cells change colour was recorded to determine the time spent in each phase of the first cell cycle following release from CDK4/6 inhibition. All images were placed on the same scale prior to analysis to ensure that the red/yellow/green cut‐offs were reproducibly calculated between experiments, which we performed using identical illumination conditions. Mitotic entry was timed based on the visualization of typical mitotic cell rounding and loss of nuclear mAG‐geminin signal.

For mitotic entry quantifications in brightfield movies, 50 cells were selected at random at the beginning of the time lapse and the time point that cells entered mitosis was determined. Mitotic entry was timed based on when the nuclear envelop breaks down and the cell rounds up.

p21 intensities were calculated for the first 100 cells in each image using ImageJ. The DAPI channel was used to generate an ROI overlay which was then applied to the p21 channel. The mean grey value of each ROI in the p21 channel was then measured along with the background intensity which was then subtracted from each of these values.

γH2AX foci were counted by eye in the first 50 cells (per condition) selected using the DAPI channel. For scoring of nuclear abnormalities, the first 100 cells within the image were counted and scored based on their nuclear morphology. 53BP1‐H2B movies were analysed by eye quantifying nuclear morphologies as mentioned above. Chromosome alignment was also scored in cells that displayed H2B expression.

To quantify EdU incorporation in different tumour lines (Fig EV4), 100 cells were randomly selected in the DAPI channel and the number of EdU‐positive cells was then counted.

## Author contributions

**Lisa Crozier:** Data curation; Formal analysis; Validation; Investigation; Visualization; Methodology; Writing – review & editing. **Reece Foy:** Data curation; Formal analysis; Validation; Investigation; Visualization; Methodology; Writing – review & editing. **Brandon L Mouery:** Data curation; Formal analysis; Validation; Investigation; Visualization; Methodology; Writing – review & editing. **Robert H Whitaker:** Data curation; Formal analysis; Validation; Investigation; Visualization; Writing – review & editing. **Andrea Corno:** Investigation. **Christos Spanos:** Investigation. **Tony Ly:** Data curation; Supervision; Funding acquisition; Investigation; Visualization; Methodology; Writing – review & editing. **Jeanette Gowen Cook:** Supervision; Funding acquisition; Writing – review & editing. **Adrian T Saurin:** Conceptualization; Data curation; Supervision; Funding acquisition; Visualization; Writing – original draft.

In addition to the CRediT author contributions listed above, the contributions in detail are:

ATS conceived the study and supervised LC and RF who performed the majority of experiments. TL designed and supervised the MS analysis that first identified reduced MCM levels, with AC and CS providing help with LC‐MS/MS sample preparation and analysis. JGC designed and supervised experiments to characterize MCM loading defects, with BLM performing the MCM FACS analysis and RHW carrying out Western analysis to quantify replisome components. ATS wrote the manuscript with comments from all authors.

## Supporting information



Appendix

Expanded View Figures PDF

Dataset EV1

Movie EV1

Movie EV2

## Data Availability

The proteomic data are available in Dataset EV1 and also uploaded to the Proteomics IDEntification Database (PRIDE), accessible at http://www.ebi.ac.uk/pride/archive/projects/PXD023435 (Project Name: CDK4/6 inhibitors induce replication stress to cause long‐term cell cycle withdrawal. Project accession: PXD023435).
