# Supplementary material for: CDK4/6 inhibitors induce replication stress to cause long‐term cell cycle withdrawal
Source: EMBO J. 2022 Jan 17;41(6):e108599. doi: 10.15252/embj.2021108599 (PMC8922273; doi:10.15252/embj.2021108599)
Supplement: Supplementary file 1 — Appendix [file EMBJ-41-e108599-s001.pdf]

Appendix Figures: Table of Contents

Appendix Figure S1: No effect of replenishing the media on cell cycle profiles observed following a prolonged palbociclib arrest.

Appendix Figure S2: Western to characterise the p53-WT and KO RPE1 and RPE1-FUCCI cells.

Appendix Figure S3: p53-dependent p21 induction during a prolonged G1 arrest.

Appendix Figure S4: Gating strategy and representative FACS profiles from the MCM loading experiment.

Appendix Figure S5: Western to characterise the p53-WT and KO MCF7 cells.

Appendix Figure S6: Low doses of palbociclib progressively increase G1 length.

Appendix Figure S7. Representative images showing genotoxic damage after 3 weeks of continuous palbociclib treatment in various tumour lines.

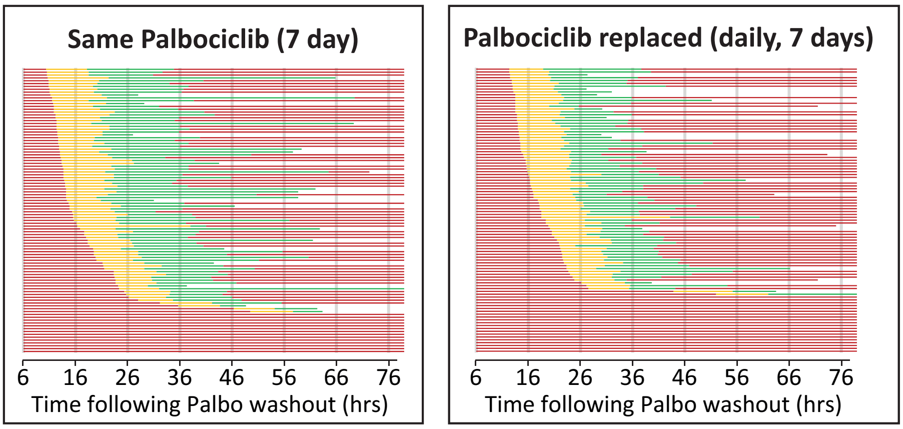

Appendix Figure S1: No effect of replenishing the media on cell cycle profiles observed following a prolonged palbociclib arrest. RPE1-FUCCI cells were treated with palbociclib (1.25 µM) for 7 days continuously (left panel) or refreshed daily with new media containing palbociclib (1.25 µM) for 7 days (right panel). Graphs show the cell cycle profile of individual RPE1-FUCCI cells (each bar represents one cell) after washout following the 7-day treatments. STLC (10 µM) was added to prevent progression through the first mitosis. 50 cells were analysed at random for each repeat and 3 experimental repeats are displayed (150 cells total).

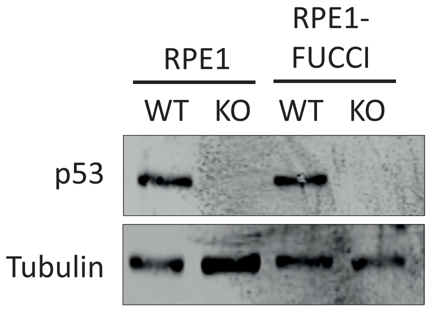

Appendix Figure S2: Western to characterise the p53-WT and KO RPE1 and RPE1-FUCCI cells. Western blot of whole cells lysates from p53-WT and KO RPE1 and RPE1-FUCCI cells treated with 50 µM Etoposide for 2 hours to induce p53, and probed with P53 or Tubulin antibodies.

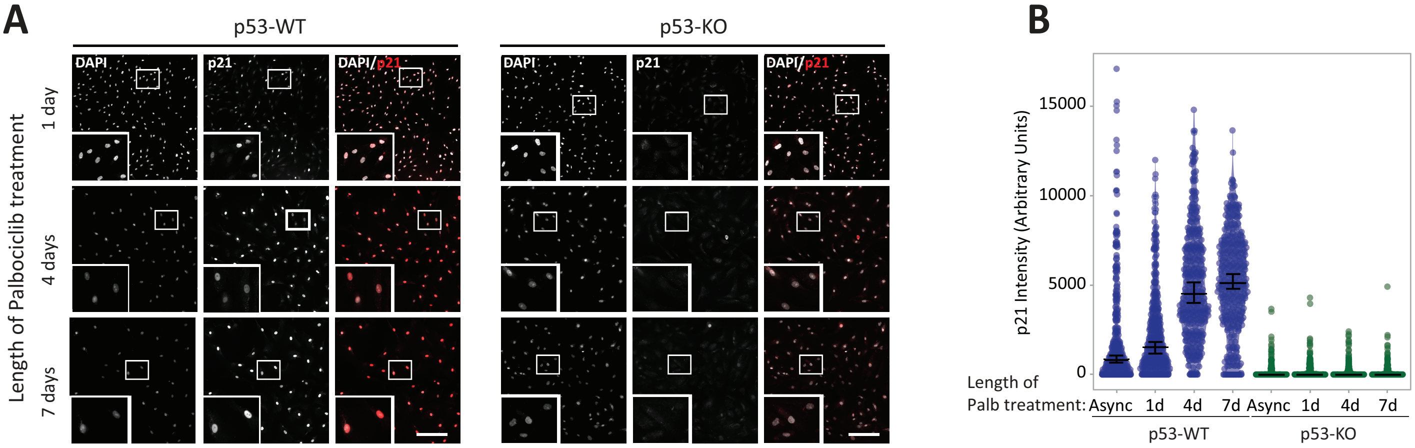

Appendix Figure S3: p53-dependent p21 induction during a prolonged G1 arrest. (A) Representative immunofluorescence images of p21 levels in p53-WT or KO RPE1 cells following 1, 4 or 7 days palbociclib (1.25µM) treatment. Zoom inserts are 3x magnification of the indicated regions. Scale bars = 250 µM. **(B)** Quantification of p21 intensities in cells treated as in panel A. At least 100 cells were analysed per experiment and graph shows data from 3 experimental repeats. Violin plots display the variation in intensities between individual cells. Horizontal lines display the median, and error bars show 95% confidence intervals.

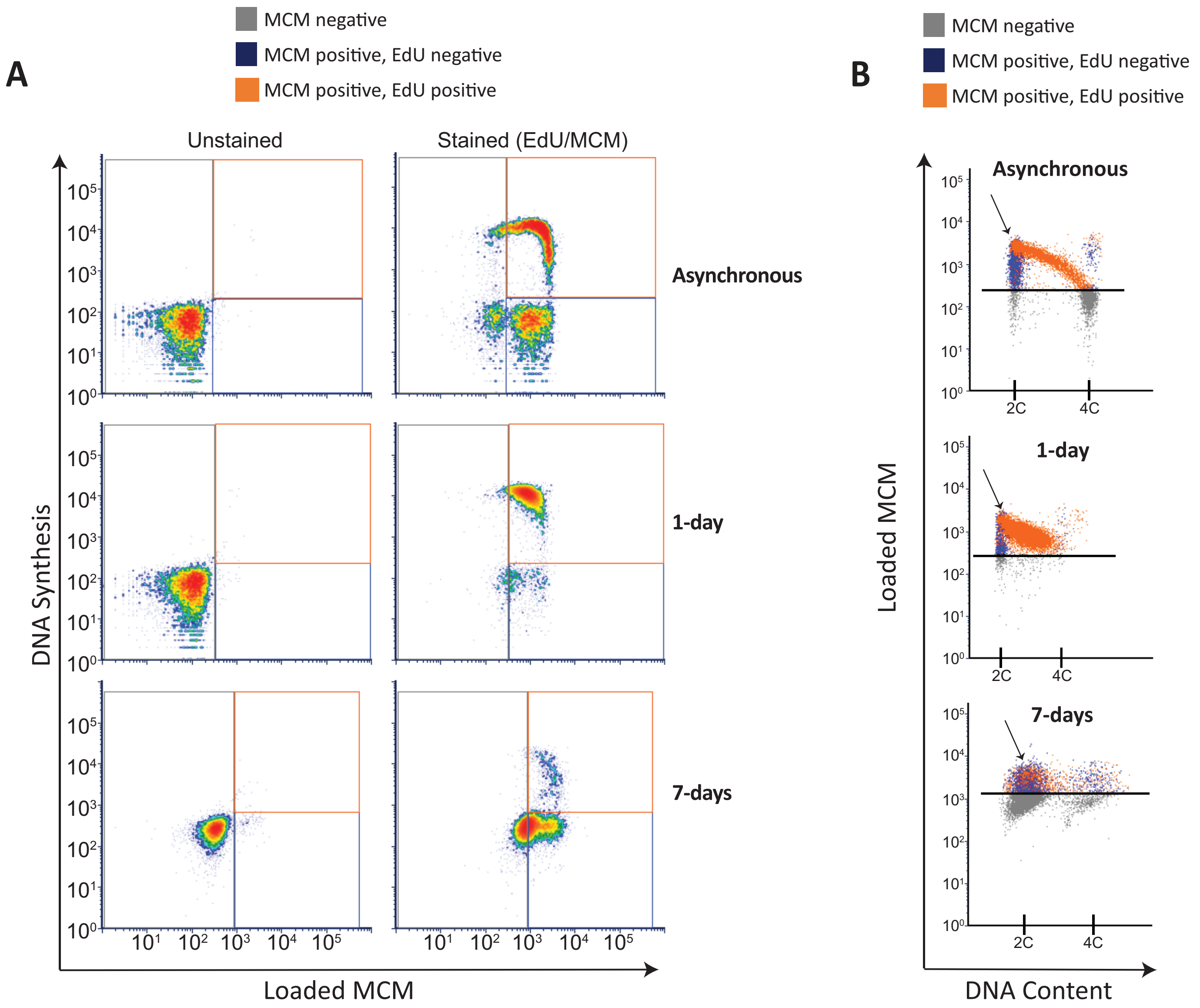

Appendix Figure S4: Gating strategy and representative FACS profiles from the MCM loading experiment. A. Gating strategy used to define cell populations. MCM intensity is shown on the x-axis and EdU intensity is shown on the y-axis. For each experimental condition, an unstained sample (left column) was used to draw gates defining MCM negative cells (grey gates on the left), MCM positive EdU negative cells (blue gates lower right), and MCM positive EdU positive cells (orange gate, upper right). These gates were then applied to samples treated identically and stained to detect MCM and EdU (right column). Due to changes in background fluorescence from cell size differences, gates were drawn differently for different experimental conditions (e.g. between 1-day and 7-day palbociclib treated cells) but the same gates were used for identically treated samples. **B.** Representative flow cytometry plots showing DNA content versus loaded MCM for samples treated as described in figure 4F. MCM positive and EdU positive cells were defined for each experimental condition using the gating strategy shown in (A). The black line on each plot marks the boundary between MCM negative cells (below line) and MCM positive cells (above line). To account for higher background fluorescence from the larger palbociclib-treated cells, data quantification in figure 4F was normalized as described in the materials and methods. The arrow on each plot marks the early S phase cells (2C DNA content, EdU positive) that were analyzed.

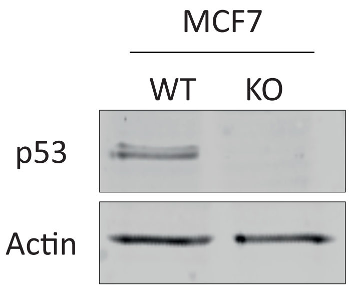

Appendix Figure S5: Western to characterise the p53-WT and KO MCF7 cells. Western blot of whole cells lysates from p53-WT and KO MCF7 cells probed with p53 or actin antibodies.

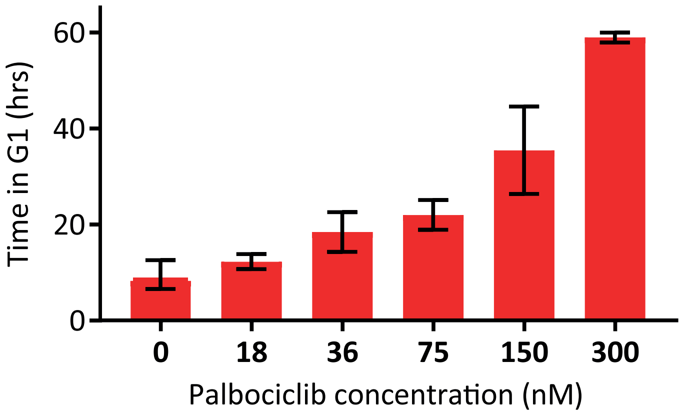

Appendix Figure S6: Low doses of palbociclib progressively increase G1 length. RPE1-FUCCI cells treated with low doses of palbociclib (0-300nM) and the length of the first G1 after division was calculated based on the duration of mKO2-Cdt1 expression. Graphs display mean data from 2 experiments, with 50 cells analysed per condition per experiment.

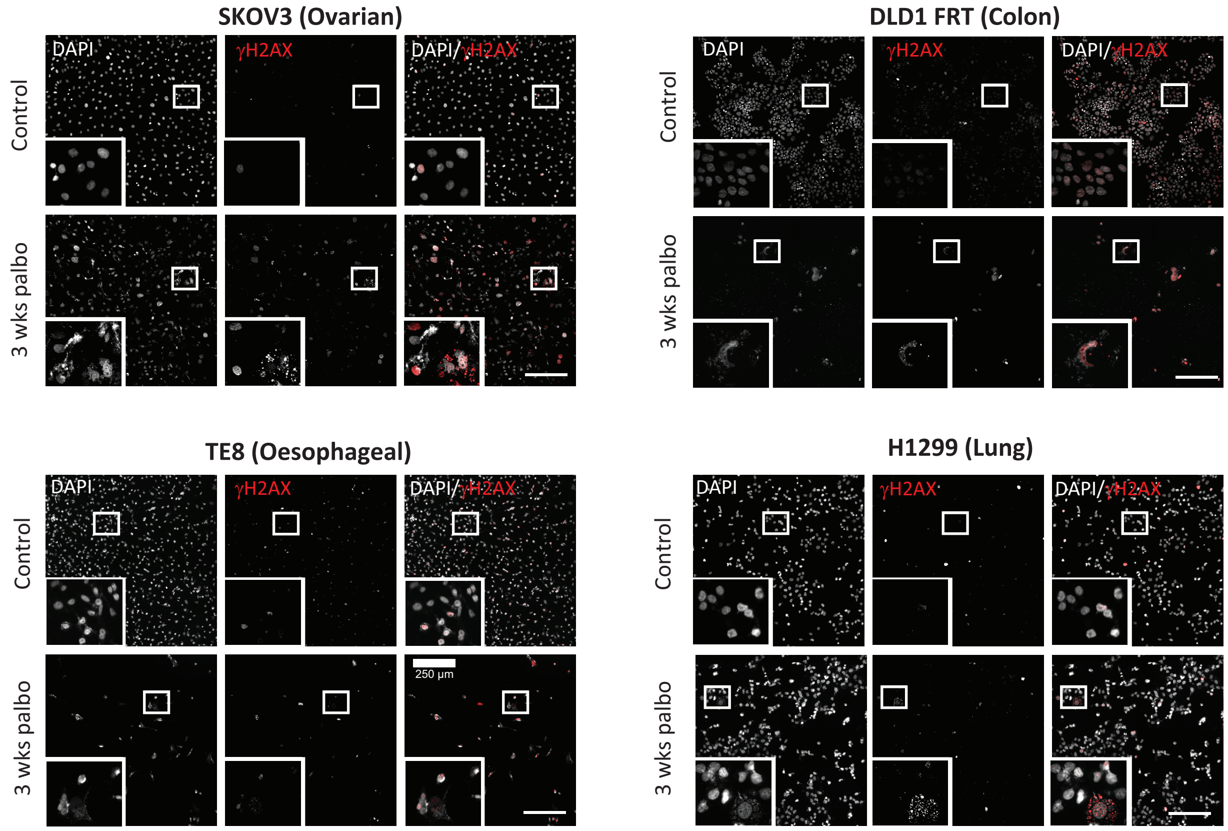

Appendix Figure S7. Representative images showing genotoxic damage after 3 weeks of continuous palbociclib treatment in various tumour lines. Representative showing nuclear morphologies (DAPI) and γH2AX-positive DNA damage foci from different tumour lines, as indicated, treated with DMSO (control) or palbociclib (1µM) for 3 weeks. Scale bars = 250µM.
