# Supplementary material for: CDK4/6 inhibitors induce replication stress to cause long‐term cell cycle withdrawal
Source: EMBO J. 2022 Jan 17;41(6):e108599. doi: 10.15252/embj.2021108599 (PMC8922273; doi:10.15252/embj.2021108599)
Supplement: Supplementary file 2 — Expanded View Figures PDF [file EMBJ-41-e108599-s004.pdf]

Expanded View Figures

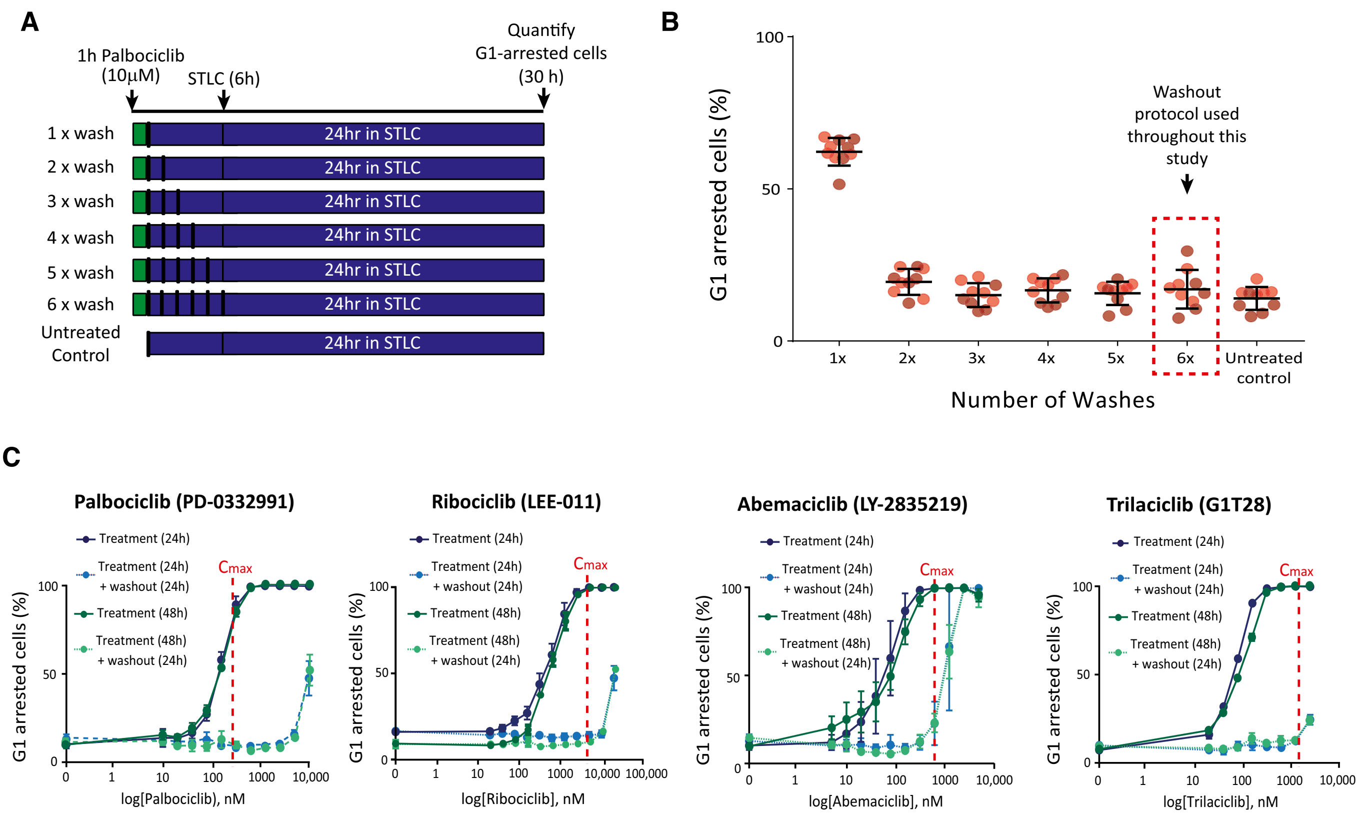

Figure EV1. Effect of drug washout protocol or inhibiting CDK4/6 for 48 h on the reversibility of the G1 arrest in RPE1 cells.

A Schematic showing different washout protocols tested to ensure washout of high-dose (10 µM) palbociclib. RPE1-FUCCI cells were treated for 1 h with 10 µM palbociclib and subsequently washed out 1–6 times, with 1 h equilibration periods interspersed between washes. STLC (10 µM) was then added to arrest cells in mitosis before quantifying the amount of mKO-Cdt1-positive, G1-arrested cells 24 h later.

B Quantification of the G1-arrested cells following the washout protocol described in (A). Graphs display the mean data ± SD from at least 500 cells counted per condition per experiments for two experimental repeats. Note, the points represent five different positions that were imaged per condition per experiment and the different coloured dots represent the two separate experiments.

C Percentage of G1-arrested RPE1-FUCCI cells after treatment for 48 h with different CDK4/6 inhibitors (dark green solid lines) or 24 h after subsequent drug washout (light green dotted lines). The data are overlaid with 24 h arrest data from Fig 1A (blue lines) to allow comparison. Vertical red dotted lines indicate C_{max} values observed in patients (taken from (He *et al*, 2017; Klein *et al*, 2018)). Graphs display mean data ± SEM from three experiments, with at least 500 cells counted per condition per experiment.

© 2022 The Authors

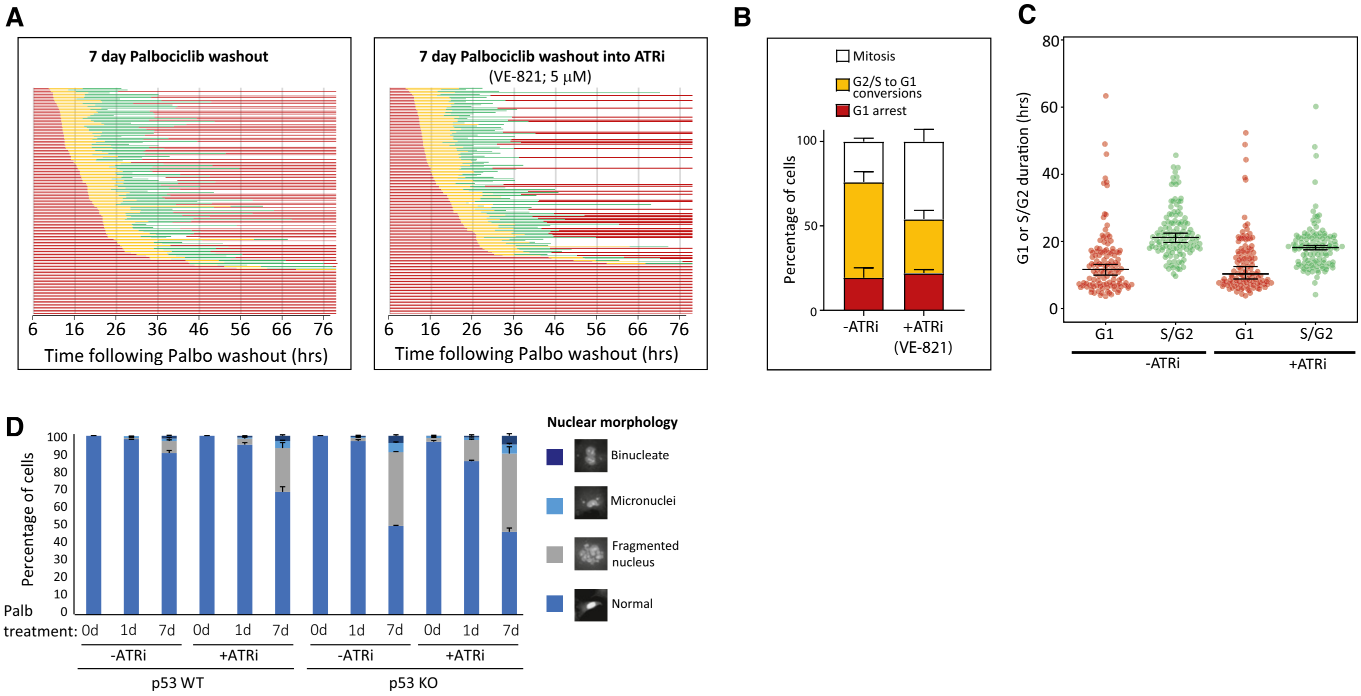

Figure EV2. Effect of ATR inhibition on the response of RPE1 cells to CDK4/6 inhibition.

A Single-cell cycle profiles quantified immediately following washout from 7 days 1.25 μM Palbociclib treatment. After washout, cells were cultured in the absence or presence of the ATR inhibitor, VE-821 (5 μM). STLC was also added at drug washout to allow analysis of just the first cell cycle. Each bar represents an individual cell, and graphs show the data from three experimental repeats (150 cells analysed in total).

B, C Quantifications of the cell cycle defects (B) and G1 or S/G2 durations (C) from the single-cell profiles shown in (A). Note, G1 length is estimated by the duration of mKO-Cdt1 expression, and S/G2 by the time from AG-Geminin expression until mitotic entry. Only cells that re-enter the cell cycle were included in this quantification (G1-arrested cells excluded). Bars graphs in (B) display mean + SD from three experimental repeats, violin plots in (C) display the variation in G1 and S/G2 length between individual cells, with horizontal lines indicating the median, and error bars representing 95% confidence intervals.

D Quantification of the nuclear morphologies following palbociclib (1.25 μM) treatment in p53-WT and KO RPE1 cells. Cells were treated for 0, 1 or 7 days and before washout for 48 h (± ATR inhibition with 5 μM VE-821). Nuclear morphologies of 100 cells were counted per condition and per experiment, and bar graphs represent mean data + SEM from three experiments.

 © 2022 The Authors

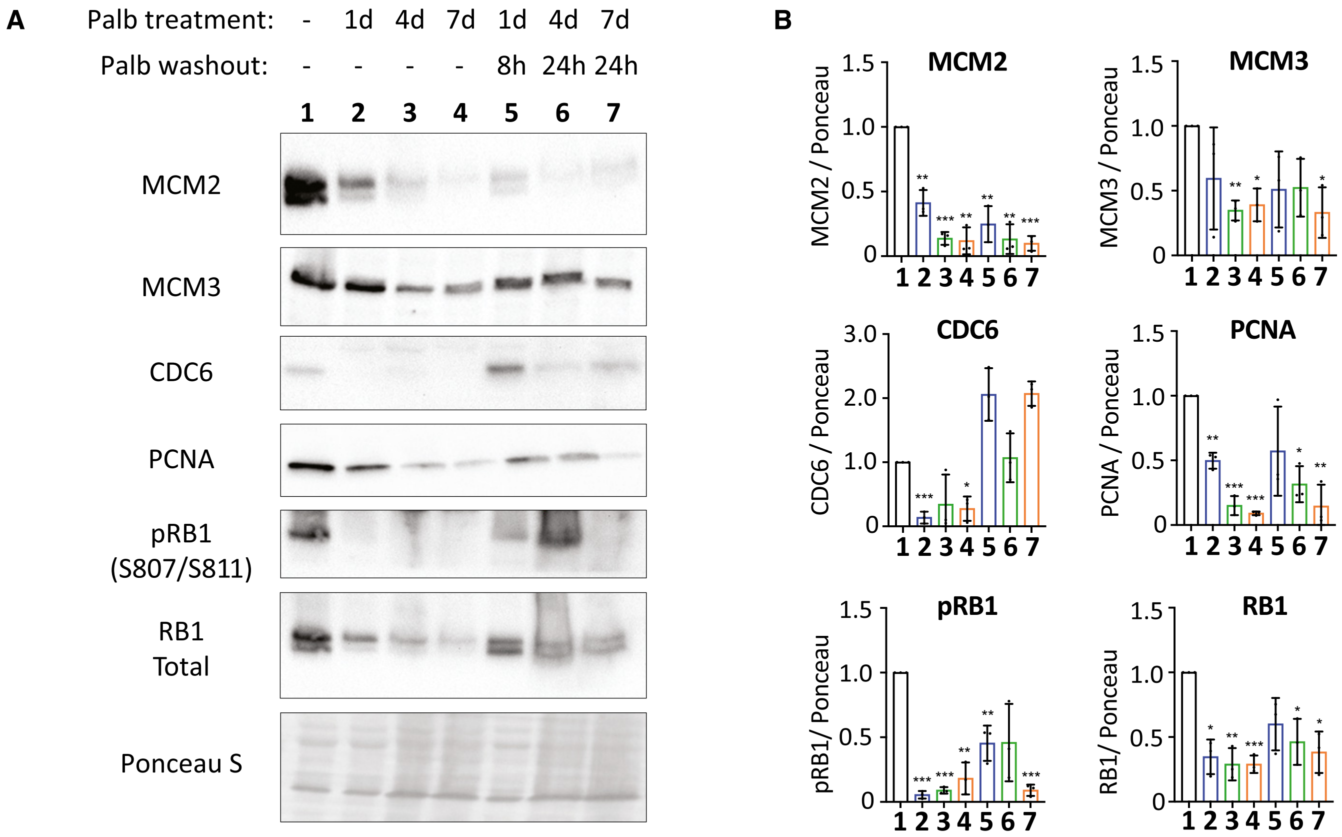

Figure EV3. Western blots to characterize the level of replisome proteins in p53-KO RPE1 cells treated with palbociclib.

A Representative western blots of whole-cell lysates from p53-KO RPE1 cells treated with palbociclib (1.25 μM) for 1, 4 or 7 days, or treated identically, and then washed out for the indicated times to reflect when the majority of cells are in S-phase (see Fig 1C).

B Analysis of adjusted relative density from three independent western blot experiments. Bars display mean values ± SD. Significance determined by unpaired Student's *t*-test comparing treated target protein to asynchronous target control (*< 0.01, **< 0.001, ***< 0.0001).

© 2022 The Authors

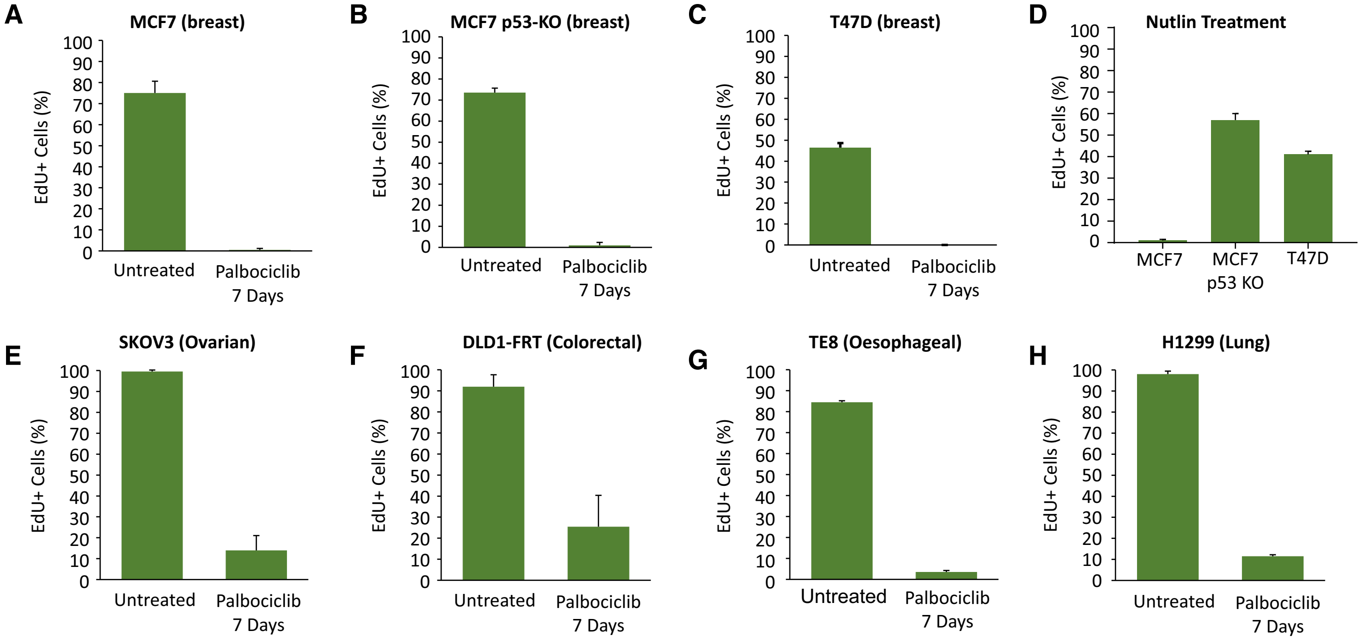

Figure EV4. EdU staining to quantify cell cycle arrest in different tumour lines treated with palbociclib or nutlin.

A–H Quantification of the percentage of cells undergoing S-phase during the final 24 h of a 7-day arrest with 1 μM palbociclib in the tumour lines indicated (A–C and E–H), or the final 24 h of a 3-day arrest with 5 μM Nutlin in the breast cancer lines indicated (D). Cells were treated with DMSO (untreated) or palbociclib (1 μM) for 7 days, or Nutlin for 3 days, with EdU (10 μM) pulsed in for the last 24 h of treatment. Data show mean + SD from two experiments, with at least 100 cells quantified per experiment.

© 2022 The Authors
