# Supplementary material for: CDK4/6 inhibitors induce replication stress to cause long‐term cell cycle withdrawal
Source: EMBO J. 2022 Jan 17;41(6):e108599. doi: 10.15252/embj.2021108599 (PMC8922273; doi:10.15252/embj.2021108599)
Supplement: Supplementary file 5 — Movie EV2 [file EMBJ-41-e108599-s006.zip › Movie EV2 legend.docx]

**Movie EV2: First division after washout from 1-day palbociclib washout in p53-KO cells.** Frames taken every 4 mins to capture first division after palbociclib (1 days, 1.25μM) washout.
