# Supplementary material for: CDK4/6 inhibitors induce replication stress to cause long‐term cell cycle withdrawal
Source: EMBO J. 2022 Jan 17;41(6):e108599. doi: 10.15252/embj.2021108599 (PMC8922273; doi:10.15252/embj.2021108599)
Supplement: Supplementary file 6 [file EMBJ-41-e108599-s002.pdf]

THE EMBO JOURNAL

CDK4/6 inhibitors induce replication stress to cause long-term cell cycle withdrawal

Lisa Crozier, Reece Foy, Brandon Mouery, Robert Whitaker, Andrea Corno, Christos Spanos, Tony Ly, Jeanette Cook, and Adrian Saurin
DOI: 10.15252/embj.2021108599

Corresponding author(s): Adrian Saurin (a.saurin@dundee.ac.uk)

Review Timeline:

Transferred from Review Commons:	28th Apr 21
Editorial Decision:	5th May 21
Revision Received:	24th Nov 21
Accepted:	21st Dec 21

Editor: Hartmut Vodermaier

Transaction Report: This manuscript was transferred to The EMBO Journal following peer review at Review Commons.

(Note: With the exception of the correction of typographical or spelling errors that could be a source of ambiguity, letters and reports are not edited. Depending on transfer agreements, referee reports obtained elsewhere may or may not be included in this compilation. Referee reports are anonymous unless the Referee chooses to sign their reports.)

1st Editorial Decision **5th May 2021**

Thank you for transferring your manuscript with Review Commons referee reports and responses to The EMBO Journal. Based on the work's potential importance and the overall interest in principle expressed by the reviewers, as well as your well-taken plans for addressing their concerns, we would be happy to consider a revision of this study further for publication in our journal. The key issue in this respect will be the extension beyond RPE1 cells towards further confirmation in (different) cancer cell lines. Regarding controversy about in vivo effects of Cdk4/6 inhibitors (G1 arrest, immune modulation), we would hope that further input from all referees and potential additional arbitrating advisors will be able to clarify this dispute within the scope of this study.

Review #1 -

Comments on 'CDK4/6 inhibitors induce replication stress to cause long-term cell cycle withdrawal'

The rationale for this work is to understand the mechanism by which Cdk4/6 inhibitors inhibit tumour cell growth, specifically via senescence which seems to be a frequent outcome of Cdk4/6 inhibition. Although several mechanisms by which Cdk4/6 inhibition induce senescence have been proposed these have varied with the cancer cell model studied. To examine the mechanism for the cytostatic effect of cdk4/6i in therapy without potential confounding effects of different cancer cell line backgrounds, Crozier et al tackle this question in the non-transformed, immortalised diploid human cell line, RPE1. They use live cell imaging and colony formation to track the impact of G1 arrests of different lengths induced by a range of clinically relevant cdk4/6 inhibitors. They also use CRISPR-mediated removal of p53 to examine the role of p53 in the observed cell cycle responses. After noting that G1 arrest of over 2 days leads to a pronounced failure in continued cell cycle and proliferation that is associated with features of replication stress, they perform a proteomics analysis to determine the factors responsible for this. They discover that MCM complex components and some other replicative proteins are downregulated and overall suggest a mechanism whereby downregulation of these essential replication components during a prolonged G1 induce replication stress and ultimate failure of proliferation. They show the impact of cdk4/6 inhibition can be increased by combining with either aneuploidy induction (to indirectly elevate replication stress), aphidicolin (to directly elevate replication stress) or chemotherapy agents that damage DNA.

Overall this is a well written and presented manuscript. Data are extremely clearly presented and described clearly within the text. Most appropriate controls were included and the work is performed to a high standard. I have a few comments about the proteomic analysis, and the link between MCM component deregulation and the induction of replication stress:

Major points:

1. Relevance to cancer. I appreciate that examining the mechanism in a diploid line is a sensible place to start. However it remains a bit unclear precisely which aspects of this mechanism might be conserved in cancer. It could be helpful to provide evidence (if it exists) of the impact of cdk4/6 inhibition in tumour cells. For example, are catastrophic mitosis, senescence, etc observed? And is there anything further known about the relationship between tumour mutations such as p53 and clinical response to Cdk4/6i? Also - many of the phenotypes followed in this manuscript vary considerably with the length of G1 and the length of release. Which of these scenarios might mimic in vivo conditions? Relating to the downregulation of MCM complex members, and the potential impact on origin licensing, how would this mechanism be manifest in cancer cells that have already deregulated gene transcription programs, and are already experiencing replication stress?
2. MCM protein levels and proposed impact on chromatin loading and origin licensing. Several MCM components are clearly reduced at the protein level. A chromatin assay (assaying fluorescence of signal remaining after pre-extraction of cytosolic proteins) suggests that MCM loading on chromatin is reduced, and this is taken to suggest a reduction in origin licensing. This is quite an indirect method - and it is difficult to conclude that the reduced chromatin bound fraction really represents a meaningful reduction in origin licensing. It

would be more convincing if either positive and negative controls for this assay were included. Moreover it is not clear if this MCM reduction and proposed reduction in licensed origins would actually impact replication in an otherwise unperturbed state? Many more origins are licensed than actually fire during a normal S-phase, so it is not entirely clear that MCM levels could lead directly to replication stress here.
3. Loss of MCM protein levels and chromatin loading occurs after 1 day, not 4 days, of Cdk4/6 inhibition. The current proposal (based on evidence from the live cell imaging, and the induction of hallmarks of replication stress in figures 1-3) seems to be that something occurs between 2 and 7 days of cdk4/6i to prevent cells from resuming a normal cell cycle. Thus the proteomics was performed between 2 and 7 days, and MCM proteins identified as major changed proteins between those times. However, according to Western blots and FACS profiles in Figure 4, the major reduction in MCM protein levels, and chromatin loading occurs already at 1 day of
of cdk4/6i (Figure 4d,e,f). However, replication stress is not observed after this timepoint (Figure 3) - so this seems to decouple the timings of MCM reduction from induction of replication stress. How can this be reconciled?

Minor points:

1. All the live cell tracking figures would be even more informative if a quantification of key features (such as a cumulative frequency of S-phase entry, or a mean+SD of time in G1, S and G2) were also presented.
2. In Figure 2D the cells released from palbociclib seem to delay longer in G1 until they start to enter S phase, compared to cells co-treated with STLC (Figure 2B). Why would this be? It is difficult to tell if other subtle effects might be present in between the +STCL and -STLC conditions, so additional graphs such as those suggested above might be informative here in particular.
3. Figure 4f It would be helpful to see the FACS plot for at least one of the conditions quantified in the graph as a comparison.
4. MCM2 protein is not down in p53 wt, but is reduced in p53 KO cells - why is this? And why is MCM2 not impacted when the other MCM complex members are?
5. Inducing aneuploidy with reversine to elevate replication stress may result in additional aneuploidy-related stresses that confound this interpretation. For example, aneuploidy per se is known to elevate p21 and p53 levels, and chromosome mis-segregation could elevate DNA damage. For these reasons these experiments are not as compelling as the direct elevation of replication stress using aphidicolin.

Interesting points to follow up/add more mechanism

1. What is mechanism of protein downregulation of MCM etc? Was gene transcription impacted, or is this a question of protein stability? Depletion of one subunit can destabilise the complex leading to protein loss of the other MCM subunits, so perhaps this effect could be due to downregulation of a single MCM complex member.
2. Are these findings specific to Cdk4/6 inhibitors, or would another means or arresting cells in G1 have the same impact?

The central question of the paper is an important one so this work would be of interest to many in the clinical and preclinical fields, and also to the cell cycle and replication stress fields.

Review #2 -

In this paper, Saurin and colleagues investigate the effects of CDK4/6 inhibitors on cell cycle arrest and re-entry. The authors report that long-term G1 arrest induced by CDK4/6i interferes with DNA replication during the next cell cycle, leading to DNA damage and mitotic catastrophe. Additionally, this compromised replication state sensitizes cells to chemotherapeutics that enhance replication stress.

The major claims advanced in this paper are well-supported by the presented evidence. Well I have several questions regarding the significance (see below), I have only a few minor points regarding the methodology.

1) Regarding the down-regulation of MCM components induced by long-term palbo treatment shown in Figure 4: MCM levels are tightly regulated by cell cycle phase. I could imagine that this gene expression change may be a consequence of, for instance, 2 days CDK4/6i treatment arresting 95% of cells in G1 while 7 days of CDK4/6i treatment causes a 99.9% G1 arrest. The data in Figure 1B seems to argue against this hypothesis, but how was that data generated? Can the authors rule out a subtle change in S-phase % over 7 days in palbo?

Alternately, is the down-regulation of MCM genes a consequence of cells entering senescence?

2) For the drug studies presented in figure 5, it is important that the authors perform the appropriate statistical comparisons and analyses to demonstrate true synergy. The authors show that combining palbo and certain chemotherapies causes a greater decrease in clonogenicity than palbo alone. This may or may not be surprising (see below) - but this by itself is insufficient to support the claim that palbo "sensitizes" cells to genotoxins. If you treat cells with two poisons, in 9 out of 10 cases, you'll kill more cells than if you treat cells with one poison alone. But that could be due to totally independent effects - see, for instance, Palmer and Sorger Cell 2017. There are several well-established statistical methods for investigating drug synergy - like Loewe Additivity or Bliss Independence - and one of these methods should be used to analyze the drug-combination studies presented in Figure 5.

While this study is a comprehensive analysis of the effects of CDK4/6i in RPE1 cells in 2d culture, I am not convinced of its broader significance.

1) So far as I can tell, the authors do not cite any studies establishing that CDK4/6i results in a significant increase in G1-arrested cells in treated patients. What evidence is there for this claim? I am aware that this has been demonstrated in xenografts and in mouse models, but I could not find evidence for this from actual clinical studies. Here, I am reminded of the very interesting work from Beth Weaver's group on paclitaxel - Zasadil STM 2014. While it had been widely assumed that paclitaxel causes a mitotic arrest, they actually show that this drug kills tumor cells by promoting mitotic catastrophe without inducing a complete mitotic arrest.

Similarly, in the absence of existing clinical data, the underlying assumption regarding the effects of CDK4/6i that motivates this paper may not be accurate. For instance, if CDK4/6i acts through the immune system (as suggested by Jean Zhao and others), then this G1 arrest phenotype could be entirely secondary to the drug's actual mechanism-of-action.

2) How relevant are RPE1 cells? Clinically, CDK4/6 inhibitors are combined with fulvestrant (which would not have an effect in RPE1), and the activity that they exhibit in breast cancer has not been matched in any other cancer types. The underlying biology of HR+ breast cancer (particularly regarding the regulation of CCND1 expression and the G1/S transition by estrogen) may not be recapitulated by other cell types. Moreover, the artificial media used in cell culture experiments may alter the regulation of the G1/S transition. I do not believe that these experiments conducted in RPE1 cells in 2d cell culture are generalizable.

3) I am confused about the effects of CDK4/6i on genotoxin sensitivity. Replogle and Amon PNAS 2020 and several citations contained therein report that CDK4/6i protects cells from DNA damage. Moreover, trilaciclib has recently received FDA approval for its ability to protect the bone marrow from cytotoxic chemotherapy! Is this a question of dose timing/intensity? The FDA approval of trilaciclib for this indication should certainly be discussed. This underscores my concern that certain findings in this paper are RPE1/tissue culture artifacts, with limited generalizability.

Referees cross-commenting

I think that we largely agree that RPE1 is not a great model for this study, and repeating certain key experiments in an ER+ BC line like MCF7 may be warranted.

Additionally, I wanted to draw attention to the fact that, to my knowledge, the evidence for palbociclib inducing a G1 arrest in patients is incredibly spotty. For early-stage breast tumors where palbo is most effective, nearly all tumor cells are in G1 anyway. I think that it makes the most sense that palbo is actually working through immune modulation or through some secondary mechanism, rather than enforcing a G1 arrest. So I'm not sure about the premise of this study.

Review #3 -

The authors clearly demonstrate, with appropriate techniques, that cells treated with clinically relevant CDK4/6 inhibitors lead to a cell cycle arrest, that is only partly reversible.

The authors also demonstrate clearly that release from a cdk4/6i arrest leads to two phenomena: the inability to initiate S-phase, and a cell cycle exit in G2.

The inability to initiate S-phase is partly dependent on p53, the cell cycle exit is fully dependent on p53.

In the absence of p53, cells that are released from a CDK4/6i block frequently enter mitosis with unrepaired DNA lesions.

The authors clearly demonstrate that cdk4/6 inhibition leads to down regulation of key

replication genes.

Combined treatment with genotoxic agents further exaggerates the phenotype of cell cycle exit upon cdk4/6 inhibition.

Specific comments:

Figure 1B: the loss of reversibility remains at approximately 50%. Does the phenotype of replication protein depletion not happen in the 50% of cells that do restart the cell cycle? it would be good if the authors could experimentally address the heterogeneity that is observed.

Figure 1C: the G1 state after S-phase. The read-out here is loss of the Fucci reporter geminin. Does observation reflect p53-dependent activation of the APC/C-Cdh1 prematerely? this is a known effect of persistent DNA damage in G2 cells.

Figure 2: there seem to be two distinct phenotypes when comparing p53-wt and p53-KO: the ability to initiate S-phase after CDK4/6i removal (which is largely gone in p53 KO, only slight number after 7d treatment). And cell cycle-drop-out after S-phase (this seems to be fully p53 dependent). I am not sure if a single mechanisms explains both.

Figure 3a: related to the proviso point. it is unclear if the p21 up regulation happens in G1 or G2 cells, and related to the inability of cells to initiate S-phase, or the cell cycle exit in G2.

It is stated that a combined action of the p53 pathways and ATR signaling prevent mitotic entry in RPE-wt cells. However, ATR should also be able to do this in p53-KO cells. Does cdk4/6i inhibiton also down-regulation of ATR pathway components?

Following the observation that CDK4/6i leads to replication stress, I would hypothesise that these cells would be very sensitive to agents that inhibit the response to replication stress (inhibitors of Wee1, ATR or Chk1). Yet, these agents work preferentially in p53-deficient cells, and require cell cycle progression. Sequential treatment with CDK4/6 inhibition followed by cell cycle checkpoint inhibition may help in uncovering the phenotype.

The authors increase the amount of replication stress using chemotherapeutic approaches or MPS1 inhibitors. The chemotherapeutic approaches are relevant clinically, but mechanistically it don't understand this beyond adding up treatments that lead to replication defects.

The aneuploidy treatment is a bit weird, because it may trigger a p53 response, before the cells are released from a cdk4.6i arrest. besides, mps1 inhibition does more than just cause replication stress and is not very clinically relevant in this context.

In their manuscript entitled: Crozier and co-workers studied the effects of CDK4/6 inhibition on cell growth. CDK4/6 inhibitors are currently used in the treatment for hormone-positive breast cancers, but their cell biological effects on tumor cells remain incompletely clear, which may hamper the further clinical development of these drugs for breast cancer or other cancers.

Inhibition of CDK4/6 is known to trigger a cell cycle arrest, and it is currently unclear how this could lead to long-term tumor control. This manuscript addresses the question why cdk4/6 inhibitors cause long-term cell cycle exit.

There are two issues that affect the significance of the findings:

-the authors start their manuscript with a strong translational/clinical issue, but solely use RPE1 cell lines to address this issue2. it remains unclear if their observations hold true in breast cancer models. it would be advised to repeat key findings in a hormone receptor-positive breast cancer model.

-the effects of CDK4/6 inhibitors are observed in clinically relevant doses. however, the effects are observed upon switch-like wash out. this does not per se reflect the pharmacodynamics of more gradual increase and decrease of drug concentrations in tuner cells. by washing out the CDK4/6 inhibitors. the significant of this work would be greater if cell cycle exit with replication stress would be observed either in clinical samples or in vivo treated cancer cells.

-the effects of CDK4/6 inhibitors are observed in clinically relevant doses. however, the effects are observed upon switch-like wash out. this does not per se reflect the pharmacodynamics of more gradual increase and decrease of drug concentrations in tuner cells. by washing out the CDK4/6 inhibitors. the significant of this work would be greater if cell cycle exit with replication stress would be observed either in clinical samples or in vivo treated cancer cells.

Response to all reviewers

We thank all the reviewers for carefully considering our manuscript and providing useful comments and suggestions. We agreed with the general comment that testing our key findings in breast cancer cells was important, and we have therefore performed extensive new analysis in the HR+ breast cancer line, MCF7, as suggested. This new data, shown in figure 6, demonstrates that replisome components are downregulated by prolonged CDK4/6 inhibition, and DNA damage and micronuclei are produced when cells are released from that arrest. This implies replication stress is induced in breast cancer cells, as it is in RPE1 cells. We also generated p53-KO MCF7s, and compared these to a p53-null HR+ breast cancer line: T47D. These data again confirm similar levels of DNA damage and micronuclei, implying replication stress is induced in different HR+ breast cancer cells. This work did reveal an interesting difference with RPE1 cells, however, because cell cycle progression was not as strongly inhibited by this replication stress and p53 played a less prominent role. There are different potential explanations for this, including that the p53 response is blunted in breast cancer cells due to MDM amplification and ARF deletion, as mentioned in out discussion. It is perhaps not surprising that cancer cells have adapted to grow better in the presence of DNA damage, in comparison to non-transformed RPE1 cells. This does highlight the importance of comparing this phenotype carefully in breast cancer cells, and therefore we believe these new experiments have been very worthwhile.

In addition to this, we elected to also look for replication stress in a variety of other tumour types, as shown in new figure 7. We did not manage to find tumour lines that could arrest as efficiently as breast cancer cells or RPE1s, therefore it was not possible to do the same 7-day treatment and drug release experiments. However, this led to the unexpected and important finding that continual CDK4/6 inhibition in these lines extends cell cycle length and causes genotoxic damage. We hypothesise that this is due to a prolonged G1 length, which we and others [1] have observed following partial CDK4/6 inhibition in RPE1 cells. Therefore, if CDK4/6 inhibition does not arrest cells fully in G1, but instead just slows G1 down, then this is also associated with elevated replication stress, but in this case during the period of continual drug treatment. This has potentially important clinical implications, since it is likely that if tumour cells proliferation is similarly only slowed down by CDK4/6 inhibition in patients, then these cells may also succumb to genotoxic damage. Therefore, this could help to induce cell cycle exit during periods of continual drug treatment. We have added a section in the discussion to highlight this point.

In addition to these major new experiments in tumour cells, we have performed additional experiments and analysis to respond to specific suggestions, as highlighted in the point-by-point responses below. We believe that these experiments have all been very worthwhile and we are glad we had this opportunity to improve our manuscript by including these at review. We therefore thank all reviewers for suggesting this new work and we hope that they will also agree that our manuscript is now greatly improved as a result.

Reviewer #1 (Evidence, reproducibility and clarity (Required)):

Comments on 'CDK4/6 inhibitors induce replication stress to cause long-term cell cycle withdrawal'

The rationale for this work is to understand the mechanism by which Cdk4/6 inhibitors inhibit tumour cell growth, specifically via senescence which seems to be a frequent outcome of Cdk4/6

inhibition. Although several mechanisms by which Cdk4/6 inhibition induce senescence have been proposed these have varied with the cancer cell model studied. To examine the mechanism for the cytostatic effect of cdk4/6i in therapy without potential confounding effects of different cancer cell line backgrounds, Crozier et al tackle this question in the non-transformed, immortalised diploid human cell line, RPE1. They use live cell imaging and colony formation to track the impact of G1 arrests of different lengths induced by a range of clinically relevant cdk4/6 inhibitors. They also use CRISPR-mediated removal of p53 to examine the role of p53 in the observed cell cycle responses. After noting that G1 arrest of over 2 days leads to a pronounced failure in continued cell cycle and proliferation that is associated with features of replication stress, they perform a proteomics analysis to determine the factors responsible for this. They discover that MCM complex components and some other replicative proteins are downregulated and overall suggest a mechanism whereby downregulation of these essential replication components during a prolonged G1 induce replication stress and ultimate failure of proliferation. They show the impact of cdk4/6 inhibition can be increased by combining with either aneuploidy induction (to indirectly elevate replication stress), aphidicolin (to directly elevate replication stress) or chemotherapy agents that damage DNA.

Overall this is a well written and presented manuscript. Data are extremely clearly presented and described clearly within the text. Most appropriate controls were included and the work is performed to a high standard. I have a few comments about the proteomic analysis, and the link between MCM component deregulation and the induction of replication stress:

- We thank the reviewer for this careful, detailed review, and for their kind comments about our work.

Major points:

1. Relevance to cancer. I appreciate that examining the mechanism in a diploid line is a sensible place to start. However it remains a bit unclear precisely which aspects of this mechanism might be conserved in cancer. It could be helpful to provide evidence (if it exists) of the impact of cdk4/6 inhibition in tumour cells. For example, are catastrophic mitosis, senescence, etc observed? And is there anything further known about the relationship between tumour mutations such as p53 and clinical response to Cdk4/6i?

- This is an important point. Whilst senescence is a common outcome of CDK4/6 inhibition in tumour cells, exactly why tumour cells become senescent is still unclear. There have been many possible explanations proposed (see introduction), but so far, none of these have implicated DNA damage. This is surprising for us, considering that DNA damage remains the best-known inducer of senescence and this is how most other broad-spectrum anti-cancer drugs induce permanent cell cycle exit. However, it was important to examine tumour cells to see if genotoxic damage and cell cycle exit can also be observed, as in RPE1 cells. This data is now included for breast cancer lines in figure 6, and in other tumour types in figure 7. The summary of this data is included in the general response to all reviewers, but the main finding is that genotoxic damage can be observed in all tumour types, implying that replication stress is a common outcome of CDK4/6 inhibition. How that stress then impacts on cell cycle progression may indeed vary between cell types and we have elaborated on this point in the discussion.

- This final point is potentially important regarding the question about p53 and clinical response. Although our RPE1 data suggests strongly that p53 loss drives resistance by preventing cell cycle exit

in response to DNA damage, this does not appear to be the case in MCF7 cells, which implies that either the p53 response is attenuated in MCF7s, or other key cell cycle control mechanisms needed to respond to DNA damage are weakened in this cell type. It will be important to examine this in future by studying the effect of p53 loss in a range of other tumour types, because P53 loss has been strongly associated with CDK4/6i resistance in the clinic, but so far this remains unexplained. Specifically, p53 loss has been implicated in abemaciclib resistance in breast cancer patients [2]), and this was recently corroborated in a larger scale study in breast cancer where p53-loss/mutation was associated with both acquired and intrinsic resistance [3]. In fact, p53 mutation/loss was the most frequent single genetic change associated with resistance in this study (58.5%), although 8 other genetic changes were also associated with resistance to differing degrees (7-27%), and these we primarily in pathways known to help drive G1/S progression following CDK4/6 inhibition (e.g. RB loss, CCNE2 amplification, RAS/AKT1 activation). Importantly, the authors could recapitulate all the resistance mechanisms in MCF7 cells, except for p53, which caused them to exclude the possibility that p53 is a driver of resistance in breast cancer. We believe there are two reasons to be cautious about that conclusion. Firstly, the resistance mechanism tested in MCF7s mainly looked at the efficiency of a G1 arrest, and this is something we do not believe is affected by p53 status. In future, it will be important to test the effect of p53 on the response to that arrest, using the types of assays we performed throughout this manuscript. Secondly, p53-KO in MCF7s did not markedly affect the long-term response to CDK4/6 inhibition, as assessed by colony forming assays (Figure 6F), although there were observable effects on the progression through the first cell cycle (Figure 6E). This could imply MCF7 have a blunted p53 response, perhaps due to the known MDM2 amplifcaiotn and p14ARF deletion, and/or that they have lost other crucial cell cycle control checkpoints that respond to DNA damage, such as ATR (Figure EV2). In future, it will be important to carefully address the role of p53 in a wide variety of cancer lines, by focusing on how it defines the response to a G1-arrest following CDK4/6 inhibition. We have now extensively discussed these points in the new manuscript.

Also - many of the phenotypes followed in this manuscript vary considerably with the length of G1 and the length of release. Which of these scenarios might mimic in vivo conditions?

- We see that a prolonged arrest (> 2 days) is necessary to see genotoxic effects in RPE cells. Clinically, palbociclib is administered in 3-week on/1-week off cycles, therefore this is consistent with the possibility that replication stress is induced during the off periods to cause genotoxic damage and cell cycle withdrawal. Importantly, our new data in figure 7 shows that genotoxic damage can also occur in tumour cells that exhibited slower cell cycle progression following CDK4/6 inhibition. Therefore, this might occur in patients during the drug-on cycles, as we elaborate on in the discussion and in the general comment to all reviewers.

Relating to the downregulation of MCM complex members, and the potential impact on origin licensing, how would this mechanism be manifest in cancer cells that have already deregulated gene transcription programs, and are already experiencing replication stress?

- We hypothesise that cancer cells with ongoing replication stress maybe more sensitive to the MCM downregulation caused by CDK4/6 inhibition. The rationale is that a reduction in licenced origins would impair the ability of dormant origins to fire in response to replication problems, therefore making elevated levels of replication stress less tolerable. This is consistent with the enhanced effect of CDK4/6 inhibition seen when replication stress is elevated in RPE cells. Moreover, others have shown that experimentally reducing MCM protein levels induces hypersensitivity to replication stress in transformed cell lines such as U2OS and HeLa [4, 5]. Thus, low MCM levels and reduced origin

licensing can contribute to replication failure in cancer cells. We have now mentioned this in the discussion.

2. MCM protein levels and proposed impact on chromatin loading and origin licensing. Several MCM components are clearly reduced at the protein level. A chromatin assay (assaying fluorescence of signal remaining after pre-extraction of cytosolic proteins) suggests that MCM loading on chromatin is reduced, and this is taken to suggest a reduction in origin licensing. This is quite an indirect method - and it is difficult to conclude that the reduced chromatin bound fraction really represents a meaningful reduction in origin licensing. It would be more convincing if either positive and negative controls for this assay were included. Moreover it is not clear if this MCM reduction and proposed reduction in licensed origins would actually impact replication in an otherwise unperturbed state? Many more origins are licensed than actually fire during a normal S-phase, so it is not entirely clear that MCM levels could lead directly to replication stress here.

- Quantifying the non-extractable MCM proteins is in truth the most direct assay for global levels of origin licensing (not origin firing) available in human cells. To our knowledge, there are no reports of MCM loading by this or similar assays that are not strongly correlated with origin licensing per se. The reviewer is correct that modest reductions in MCM loading are well-tolerated in the absence of other perturbations. Specifically, Ge et al found no proliferation effects after 50% MCM loading reduction, but any further reduction introduced a proliferation delay [5]. Of note, the U2OS cells used in that study also have a functional p53 response.

- Another important point that is worth emphasizing, is that many of the differentially downregulated proteins only function at replication forks (fig.4c). Therefore, we believe that the replication stress is a combined result of poor licencing and reduced levels of replication fork proteins that are needed after the origins fire in S-phase. We have clarified this point in the revised manuscript.

3. Loss of MCM protein levels and chromatin loading occurs after 1 day, not 4 days, of Cdk4/6 inhibition. The current proposal (based on evidence from the live cell imaging, and the induction of hallmarks of replication stress in figures 1-3) seems to be that something occurs between 2 and 7 days of cdk4/6i to prevent cells from resuming a normal cell cycle. Thus the proteomics was performed between 2 and 7 days, and MCM proteins identified as major changed proteins between those times. However, according to Western blots and FACS profiles in Figure 4, the major reduction in MCM protein levels, and chromatin loading occurs already at 1 day of
of cdk4/6i (Figure 4d,e,f). However, replication stress is not observed after this timepoint (Figure 3) - so this seems to decouple the timings of MCM reduction from induction of replication stress. How can this be reconciled?

- We agree that some of the observed changes to replisome components are quite considerable after just 1 day of arrest (some of these downregulations such as Cdc6 or phospho-Rb can be attributed to the cell cycle arrest itself - Cdc6 is unstable in G1 - but others, such MCM proteins, are not typically lost during G1). We were initially surprised by this too, considering that the phenotype clearly appears later than 1 day of arrest. It is important to state though, that the levels of almost all replisome components continue to decline as the duration of arrest is extended, eventually falling to considerably lower levels than seen after just 1 day. This is observed for MCM2, MCM3 and PCNA by western (fig.4d,e) and a large number of other replisome components by proteomics (fig.4c, 2 vs 7 days). Even MCM loading, which is 58% reduced after just 1-day arrest, is still reduced even further to

just 20% of controls after 7 days (p<0.0004). Therefore, things are clearly not normal after 1 day of an arrest, but they are much worse as that arrest progresses for longer. This is also broadly recapitulated in the new data from MCF7 cells (figure 6A,B).

Our interpretation of the phenotypic data in light of this, is that replication problems become apparent when the number of licensed origins and the function of the replisome is compromised below a certain threshold; which most likely depends on cell type and, in particular, the levels of endogenous replication stress. So, in RPE1 cells, 1-day treatment is clearly tolerable, perhaps because there are still enough origins to complete DNA replication successfully. But, importantly, if replication stress is enhanced in these cells then 1-day of palbociclib arrest now starts to cause observable defects. This is evident in Figure 5g, where 1-day palbociclib treatment causes minimal effect on long-term growth on its own, but growth is reduced considerably when replication stress is elevated with genotoxic drugs. We interpret this to mean that the reduction in licenced origins and replisome components observed after 1 day of arrest, starts to become problematic in situations when replication stress is elevated. In MCF7 cells, we observed enhanced micronuclei and γH2AX foci after release from just 1-day of palbociclib treatment (figure 6c,d) , further implying that replisome downregulation during 1-day of arrest is sufficient to evaluate replication stress. These important points have now been highlighted in the discussion.

Minor points:

1. All the live cell tracking figures would be even more informative if a quantification of key features (such as a cumulative frequency of S-phase entry, or a mean+SD of time in G1, S and G2) were also presented.

- We agree that this will be useful, and we have now included new graphs to quantify the main phenotypes of G1 arrest, G1 delays and G2>S conversions. These are included next to the single cell profiles in all relevant figures.

2. In Figure 2D the cells released from palbociclib seem to delay longer in G1 until they start to enter S phase, compared to cells co-treated with STLC (Figure 2B). Why would this be? It is difficult to tell if other subtle effects might be present in between the +STCL and -STLC conditions, so additional graphs such as those suggested above might be informative here in particular.

- Fig.2d shows only representative experiment (50 cells) because it is difficult to interpret these individual cell cycle profiles when more than 50 cells are presented. However, we have now quantified all the data from 3 experiments (150 cells) and added this to figure 2. The is a small delay (few hrs) in cell cycle re-entry in comparison to cells treated without STLC, but we do not observe any other clear differences. We cannot easily explain the delayed G1 exit, except that when looking at the movies the cells appear to be more dense that our usual experiments. We were careful to maintain a low cell density in all of these experiments to prevent cell-cell contact from affecting exit from G1 (a point we have now clarified in the main text and methods). Whilst we did carefully control cell number in these experiments, sometimes the movies are taken from positions in which the cells are more densely packed. This appear to be the case in the experiment without STLC, so we can only think that perhaps this caused a small additional G1 delay in these experiments.

3. Figure 4f It would be helpful to see the FACS plot for at least one of the conditions quantified in the graph as a comparison.

- Examples FACS plots for all conditions have now been included in Appendix Figure S4.

4. MCM2 protein is not down in p53 wt, but is reduced in p53 KO cells - why is this? And why is MCM2 not impacted when the other MCM complex members are?

- MCM2 is downregulated by palbociclib in both WT and p53KO cell lines, but the reviewer is correct that there is a small difference in MCM2 levels after 1 day of treatment ~59% remaining at day 1 in WT cells, and ~41% remaining at day 1 in the p53 null cells; both cell lines show further reductions at days 4 and 7. In addition, MCM 3 is also downregulated in both cell lines (Figure 4d and 4e and Figure EV3). We have observed some variation in the degree of downregulation among different MCM subunits, but the explanation for that difference will require us to define the mechanism(s) of downregulation.

5. Inducing aneuploidy with reversine to elevate replication stress may result in additional aneuploidy-related stresses that confound this interpretation. For example, aneuploidy per se is known to elevate p21 and p53 levels, and chromosome mis-segregation could elevate DNA damage. For these reasons these experiments are not as compelling as the direct elevation of replication stress using aphidicolin.

- We agree that the aneuploidy experiment could have many different interpretations, and only one of these relates specifically to replication stress. This was also commented on by reviewer 3, so we feel it is best to remove this data and just keep the data on drugs that affect replication stress or DNA damage directly. We will address the effects of aneuploidy more extensively in a separate study.

Interesting points to follow up/add more mechanism

1. What is mechanism of protein downregulation of MCM etc? Was gene transcription impacted, or is this a question of protein stability? Depletion of one subunit can destabilise the complex leading to protein loss of the other MCM subunits, so perhaps this effect could be due to downregulation of a single MCM complex member.
2. Are these findings specific to Cdk4/6 inhibitors, or would another means or arresting cells in G1 have the same impact?

- Both of these points are interesting questions and they are actually the focus of an entirely separate study that is ongoing. In particular, we are working on the mechanism(s) of MCM and replisome downregulation.

Reviewer #1 (Significance (Required)):

The central question of the paper is an important one so this work would be of interest to many in the clinical and preclinical fields, and also to the cell cycle and replication stress fields.

- We thank the reviewer for this, and we agree that linking CDK4/6 inhibitors to genotoxic stress is important both for our understanding of cell cycle control and for cancer treatment. We are actually very surprised that these drugs have not previously been linked to genotoxic stress, given that they appear to have broad pan-cancer activity and all other broad-spectrum anti-cancer drug work by

causing genotoxic stress.

Reviewer #2 (Evidence, reproducibility and clarity (Required)):

In this paper, Saurin and colleagues investigate the effects of CDK4/6 inhibitors on cell cycle arrest and re-entry. The authors report that long-term G1 arrest induced by CDK4/6i interferes with DNA replication during the next cell cycle, leading to DNA damage and mitotic catastrophe. Additionally, this compromised replication state sensitizes cells to chemotherapeutics that enhance replication stress.

The major claims advanced in this paper are well-supported by the presented evidence. Well I have several questions regarding the significance (see below), I have only a few minor points regarding the methodology.

1) Regarding the down-regulation of MCM components induced by long-term palbo treatment shown in Figure 4: MCM levels are tightly regulated by cell cycle phase. I could imagine that this gene expression change may be a consequence of, for instance, 2 days CDK4/6i treatment arresting 95% of cells in G1 while 7 days of CDK4/6i treatment causes a 99.9% G1 arrest. The data in Figure 1B seems to argue against this hypothesis, but how was that data generated? Can the authors rule out a subtle change in S-phase % over 7 days in palbo?

Alternately, is the down-regulation of MCM genes a consequence of cells entering senescence?

- We have performed extensive long-term movies with these cells, and we never see cells dividing or exiting G1 after the first day of palbociclib treatment. This is illustrated in figure 1b which demonstrates that 100% of FUCCI cells are in G1 (Red/mKO-Cdt1-positive) at each timepoint. This was calculated using quantitative microscopy before/after drug release in at least 500 cells per condition per experiment. This has now been clarified in the legends and methods. In addition, MCM protein levels do not actually oscillate with cell cycle phase [6, 7], although their mRNA levels certainly do [8, 9]. Furthermore, RPE and mammalian fibroblasts retain MCM proteins after 2 days of growth factor withdrawal despite transcriptional repression of their respective genes [10, 11]

- We see significant changes in MCM levels at a time when cells are still permissive to enter the cell cycle following drug release. Therefore, MCM reduction is not a consequence of senescence. Rather, we believe that it is one of the causes of cell cycle withdrawal following the subsequent S-phase.

2) For the drug studies presented in figure 5, it is important that the authors perform the appropriate statistical comparisons and analyses to demonstrate true synergy. The authors show that combining palbo and certain chemotherapies causes a greater decrease in clonogenicity than palbo alone. This may or may not be surprising (see below) - but this by itself is insufficient to support the claim that palbo "sensitizes" cells to genotoxins. If you treat cells with two poisons, in 9 out of 10 cases, you'll kill more cells than if you treat cells with one poison alone. But that could be due to totally independent effects - see, for instance, Palmer and Sorger Cell 2017. There are several well-established statistical methods for investigating drug synergy - like Loewe Additivity or Bliss Independence - and one of these methods should be used to analyze the drug-combination studies presented in Figure 5.

- The methods for statistically analysing drug synergy, such as Chou-Talalay methodology (combination indices), would require an extensive set of new data to be generated, including equimolar drug dilution titrations of the monotherapies and drug combinations. We believe this is beyond the scope of this study. Furthermore, we do not claim the drug combinations are synergistic, only that they combine to lead to enhanced cell cycle exit. For clarity, we have reworded the manuscript to state that there is increased sensitivity to the drug combination compared to the monotherapies. Future studies can be designed to assess synergy to a range of cytotoxic drug combinations in different cancer lines.

Reviewer #2 (Significance (Required)):

While this study is a comprehensive analysis of the effects of CDK4/6i in RPE1 cells in 2d culture, I am not convinced of its broader significance.

1) So far as I can tell, the authors do not cite any studies establishing that CDK4/6i results in a significant increase in G1-arrested cells in treated patients. What evidence is there for this claim? I am aware that this has been demonstrated in xenografts and in mouse models, but I could not find evidence for this from actual clinical studies. Here, I am reminded of the very interesting work from Beth Weaver's group on paclitaxel - Zasadil STM 2014. While it had been widely assumed that paclitaxel causes a mitotic arrest, they actually show that this drug kills tumor cells by promoting mitotic catastrophe without inducing a complete mitotic arrest. Similarly, in the absence of existing clinical data, the underlying assumption regarding the effects of CDK4/6i that motivates this paper may not be accurate. For instance, if CDK4/6i acts through the immune system (as suggested by Jean Zhao and others), then this G1 arrest phenotype could be entirely secondary to the drug's actual mechanism-of-action.

- We are very surprised by the suggestion that CDK4/6 inhibitors may not need to cause a G1 arrest in patient tumours. We appreciate that that these inhibitors affect the immune system in many different ways to combat tumourigenesis, but there is also an overwhelming amount of evidence that a G1-arrest in patient tumours is critical for the overall response. Perhaps the most striking evidence is the fact that RB loss in tumours is one of the best-characterised mechanism of resistance in breast cancer patients [3, 12-15]. This is also the case for CDK2 hyperactivity and Cyclin E overexpression, which drives cells through G1 independently of CDK4/6. In addition, tumour types that typically achieve a poor CDK4/6i-induced G1 arrest in preclinical models, such as TNBC, also exhibit a poor response to CDK4/6i therapy in patients. Recently a luminal androgen receptor subtype of TNBCs has been identified that responds to CDK4/6 inhibition, due to low CDK2 activity which can otherwise drive G1 progression independently of CDK4/6 in basal-like TNBCs [16, 17]. This rationalises combination therapies that converge to inhibit G1 more effectively in this subtype (e.g. AR antagonist + CDK4/6 inhibition [18]), which is akin to the oestrogen receptor and CDK4/6 combinations that have proven so successful at treating HR+ breast cancer. Many other combinations are also currently in trials based on the same premise that inhibiting upstream G1/S regulators can enhance the response by inducing a more efficient G1 arrest (MEK, PI3K, AKT, mTOR) [19].

- In response to the specific question about clinical G1 arrest in patients, tumour samples from breast cancer patients have demonstrated a decrease in the S-phase specific markers pRB and TopoIIa following abemaciclib treatment [2] and there is extensive evidence of a profound cell cycle arrest following CDK4/6 inhibition, as judged by staining with the mitotic marker Ki67 [20-23]. Whilst this

does not formally prove a G1-arrest is specifically responsible for this overall cell cycle arrest in patients, that is the implicit assumption given the known mechanism of action of CDK4/6 inhibitors in cells.

2) How relevant are RPE1 cells? Clinically, CDK4/6 inhibitors are combined with fulvestrant (which would not have an effect in RPE1), and the activity that they exhibit in breast cancer has not been matched in any other cancer types. The underlying biology of HR+ breast cancer (particularly regarding the regulation of CCND1 expression and the G1/S transition by estrogen) may not be recapitulated by other cell types. Moreover, the artificial media used in cell culture experiments may alter the regulation of the G1/S transition. I do not believe that these experiments conducted in RPE1 cells in 2d cell culture are generalizable.

- Fulvestrant/tamoxifen are effective because they enhance the efficiency of a CDK4/6i arrest by reducing Cyclin D expression to enhance Cyclin D-CDK4/6 inhibition. That convergence onto the G1/S transition is why ER antagonists enhance the CDK4/6 response. i.e. CDK activity is inhibited and CycD transcription is reduced, therefore this double hit allows breast cancer cells to arrest in G1 more efficiently than healthy tissue which is not oestrogen-responsive (this provides yet more evidence the G1 arrest in tumours is crucial for the clinical response). It is true that RPE1 cells do not respond to the oestrogen treatment, but that is not really relevant here in our opinion. We are not testing the efficiency of a G1 arrest beyond the initial characterisation in figure 1. We are mainly examining how cells respond to that G1 arrest afterwards. It could be that components of the cell culture media affect that downstream response in unanticipated ways, but we feel that is very unlikely.

- Having said that, we do agree with the general point that questions the relevance of data from just RPE1 cells. We have therefore now repeated key experiments in different breast cancer lines and in a range of other tumour types. The new data, which is summarised in the general response to all reviewers, demonstrates that the induction of genotoxic stress following CDK4/6 inhibition is conserved in tumour cells.

3) I am confused about the effects of CDK4/6i on genotoxin sensitivity. Replogle and Amon PNAS 2020 and several citations contained therein report that CDK4/6i protects cells from DNA damage. Moreover, trilaciclib has recently received FDA approval for its ability to protect the bone marrow from cytotoxic chemotherapy! Is this a question of dose timing/intensity? The FDA approval of trilaciclib for this indication should certainly be discussed. This underscores my concern that certain findings in this paper are RPE1/tissue culture artifacts, with limited generalizability.

- The studies the reviewer refers to demonstrate that halting cell cycle progression can protect cells from genotoxic drugs that cause DNA damage during S-phase. However, we can only think that the reviewer must have missed the critical point here: The genotoxic agents in figure 5 were added <u>after washout</u> from the CDK4/6 inhibitor (we have highlighted this more clearly in the revised manuscript). After release from CDK4/6 inhibition, cells enter S-phase with replication problems as a result of the G1 arrest/replisome downregulation, and they then experience additional problems during S-phase, as a result of the genotoxic agents included following the washout. These effects combine to enhance replication stress, a key conclusion of figure 5.

- The fact that trilaciclib is now licenced for bone marrow protection is discussed in the new manuscript. It should be pointed out though that this was licenced in SCLC which are almost all Rb-null - this prevent CDK4/6 inhibitors from arresting the tumour cells and also invertedly protecting

them from chemotherapeutic damage. Interestingly, preliminary data from similar trial in triple-negative breast cancer could not demonstrate myeloprotection with trilaciclib, but in these patients there was a marked improval in overall survival in the triliciclib + genotoxic drug arms. In this case, the genotoxic drug was given either together with, or immediately after CDK4/6 inhibitor (as in out experimental setup). Therefore. it is possible that this enhance response could be caused by the ability of CDK4/6 to enhance DNA damage in tumour cells. We have updated the discussion to include this point.

- There are also large number of other studies that demonstrate the benefit of combining cytotoxic chemotherapeutic with CDK4/6 inhibitors, so these combinations are not only useful for providing myeloprotection. This is discussed in detail in this review [24], and we have now extensively discussed this point in the revised manuscript. We believe our manuscript could provide a new and important mechanistic explanation for these synergistic drug combinations.

- These prior studies on trilaciclib in no way support that notion that "findings in this paper are RPE1/tissue culture artefacts with limited generalizability". Experiments in 2D tissue culture have furnished some of the most important fundamental discoveries in cancer research. It remains to be seen whether our study will cause a paradigm shift in our thinking about how CDK4/6 inhibitors work, but we believe that it may do. We appreciate that this will not become clear until our findings are followed up and validated in preclinical models and human disease, but that does not, in our opinion, make them any less valid at this stage. As stated earlier, we have now confirmed that this is not a RPE1 cell phenomenon, showing that the initial results hold true in breast cancer cells and in other tumour types. Therefore, we believe our data will have an important impact on future preclinical and clinical work in this area.

Referees cross-commenting

I think that we largely agree that RPE1 is not a great model for this study, and repeating certain key experiments in an ER+ BC line like MCF7 may be warranted.

- We have now repeated key experiments in MCF7 -/+ p53 cells and in the p53-null T47D, which demonstrates that key result on replisome downregulation and genotoxic damage after S-phase hold true. We have also observed damage in a range of other tumour types treated with CDK4/6 inhibitor.

Additionally, I wanted to draw attention to the fact that, to my knowledge, the evidence for palbociclib inducing a G1 arrest in patients is incredibly spotty. For early-stage breast tumors where palbo is most effective, nearly all tumor cells are in G1 anyway. I think that it makes the most sense that palbo is actually working through immune modulation or through some secondary mechanism, rather than enforcing a G1 arrest. So I'm not sure about the premise of this study.

- As discussed above, there is extensive evidence that proliferation is reduced in response to CDK4/6 inhibition in patients [2, 20-23]. We agree that a smaller proportion of cells are proliferative in patient tumours compared to preclinical models, and there can be many reasons for this, especially within solid tumour where hypoxia is a major factor that limits proliferation. However, we do not agree that this implies that drugs that target these tumours do not act on the proliferating cells. In fact, most other broad-spectrum non-targeted chemotherapies used to treat cancer also work by targeting dividing cells, and many of these are also effective in breast cancer. In addition, and as discussed extensively above, there are many studies supporting the interpretation that a G1 arrest is

critical for CDK4/6i response in breast cancer patients. Considering all of these points, we strongly believe that the premise of our study – to characterise why a G1 arrest becomes irreversible – is valid and important. This point is also made in numerous recent reviews which also highlight that this key mechanistic information is currently lacking [19, 25-27].

- We agree with the idea that the immune effects are also important in patients, and indeed, we cited and discussed these studies in our manuscript. However, we would argue that this works together with a G1 arrest in tumour cells. The G1 arrest most likely induces a senescent response that stimulates immune engagement and tumour clearance. These combined effects of CDK4/6 inhibitors, on both the tumour and the immune system, are discussed at length in these reviews: [19, 25, 27].

Reviewer #3 (Evidence, reproducibility and clarity (Required)):

The authors clearly demonstrate, with appropriate techniques, that cells treated with clinically relevant CDK4/6 inhibitors lead to a cell cycle arrest, that is only partly reversible.

The authors also demonstrate clearly that release from a cdk4/6i arrest leads to two phenomena: the inability to initiate S-phase, and a cell cycle exit in G2.

The inability to initiate S-phase is partly dependent on p53, the cell cycle exit is fully dependent on p53.

In the absence of p53, cells that are released from a CDK4/6i block frequently enter mitosis with unrepaired DNA lesions.

The authors clearly demonstrate that cdk4/6 inhibition leads to down regulation of key replication genes.

Combined treatment with genotoxic agents further exaggerates the phenotype of cell cycle exit upon cdk4/6 inhibition.

Specific comments:

Figure 1B: the loss of reversibility remains at approximately 50%. Does the phenotype of replication protein depletion not happen in the 50% of cells that do restart the cell cycle? It would be good if the authors could experimentally address the heterogeneity that is observed.

- This is actually a result of the fixed analysis used in figure 1B. The irreversibility is much higher than 50% after long durations of arrest, but at the 24h timepoint used in this fixed assay many cells have exited G1 but not yet had a chance to revert back into G1 from S/G2 phase. We have now clarified the details of this assay in the revised manuscript. This highlights the value of our extensive live cell assays that can fully capture cell cycle profiles, and accurately determine when cells do/don't enter or withdraw from different stages of the cell cycle. We believe that an overreliance of fixed endpoints in previous studies may have contributed to the genotoxic effects in S-phase being missed previously: many studies show senescence after drug washout, but the cause of that senescence only becomes apparent when you observe that cells withdraw with defects after the first S-phase.

Figure 1C: the G1 state after S-phase. The read-out here is loss of the Fucci reporter geminin. Does observation reflect p53-dependent activation of the APC/C-Cdh1 prematerely? this is a known effect of persistent DNA damage in G2 cells.

- Yes, we expect that APC/C-Cdh1 activation causes geminin and cyclin degradation when cells permanently withdraw from the cell cycle from G2. This is likely caused by p53-dependent p21 activation in response to DNA replication defects, as has been shown previously in direct response to DNA damage.

Figure 2: there seem to be two distinct phenotypes when comparing p53-wt and p53-KO: the ability to initiate S-phase after CDK4/6i removal (which is largely gone in p53 KO, only slight number after 7d treatment). And cell cycle-drop-out after S-phase (this seems to be fully p53 dependent). I am not sure if a single mechanisms explains both.

- We agree that there are p53-dependent effects on speed/extent of S-phase entry and on the resulting withdrawal from G2. It may not be a single mechanism that connected these effects, although they may be related. Our manuscript mainly focusses on the DNA replication defects and cell cycle withdrawal, but in the future, it will be important to also characterise what causes the delay in cell cycle re-entry following CDK4/6 inhibition. We suspect that this could reflect differing depths of quiescence, potentially caused by p21, which would explain the p53-dependence.

Figure 3a: related to the proviso point. it is unclear if the p21 up regulation happens in G1 or G2 cells, and related to the inability of cells to initiate S-phase, or the cell cycle exit in G2.

- This is a good point, and as discussed above, we suspect both maybe related to p21. We performed analysis of p21 during the G1 arrest and observed a increase in p21 levels upon prolonged arrest. This data is now included in appendix figure S3.

It is stated that a combined action of the p53 pathways and ATR signaling prevent mitotic entry in RPE-wt cells. However, ATR should also be able to do this in p53-KO cells. Does cdk4/6i inhibiton also down-regulation of ATR pathway components?

- We do not detect downregulation of any ATR components in the mass spec data comparing 2 and 7 day palbociclib arrest.

Following the observation that CDK4/6i leads to replication stress, I would hypothesise that these cells would be very sensitive to agents that inhibit the response to replication stress (inhibitors of Wee1, ATR or Chk1). Yet, these agents work preferentially in p53-deficient cells, and require cell cycle progression. Sequential treatment with CDK4/6 inhibition followed by cell cycle checkpoint inhibition may help in uncovering the phenotype.

- This is a good point and we have performed extensive experiments with ATR inhibitors after release from CDK4/6 inhibition to examine if this enhances the phenotype. The new data in now included in figure EV2 and this shows that ATR inhibition increases the fraction of p53-WT cells that reach mitosis following washout from prolonged CDK4/6 inhibition. This is associated with an increase in fragmented nuclei, implying that ATR does indeed guard against replication stress by promoting cell cycle withdrawal.

The authors increase the amount of replication stress using chemotherapeutic approaches or MPS1 inhibitors. The chemotherapeutic approaches are relevant clinically, but mechanistically it don't understand this beyond adding up treatments that lead to replication defects.

- We agree that the main value of these experiments is not to provide mechanistic insight, but rather to demonstrate that CDK4/6 inhibition can enhance the effect of current genotoxic drugs (when applied immediately before these drugs). Considering CDK4/6 inhibitors are well-tolerated, this could represent an effective way to enhance the tumour-selectivity of current genotoxic therapeutics. This has been documented in various other preclinical studies, which have guided recent clinical trials to assess these novel combinations. This is discussed extensively in this review {Roberts, 2020 #1225}, and we have now written an expanded section in the discussion to highlight these points.

- Although our drug combination experiments do not explore mechanism, we believe these put forward a new idea that could explain the synergistic effects observed by others. They imply that CDK4/6i and genotoxic drugs converge onto the same replication stress phenotype, thereby supporting our overall conclusions. One interpretation of these effects, is that a reduction in replisome levels and licenced replication origins impairs the ability of cells to overcome replication problems induced by chemotherapy drugs. This is a hypothesis that has not been discussed previously, which is important, because conceptualising how these genotoxic drugs might be sensitized by CDK4/6 inhibition will be important in designing new studies and trials to address whether these combinations are broadly synergistic.

The aneuploidy treatment is a bit weird, because it may trigger a p53 response, before the cells are released from a cdk4.6i arrest. besides, mps1 inhibition does more than just cause replication stress and is not very clinically relevant in this context.

- We agree that the aneuploidy experiment could have many different interpretations, and only one of these relates specifically to replication stress. This was also commented on by reviewer 1, so we have removed this data and just keep the data on drugs that affect replication stress or DNA damage directly. We will address the effects of aneuploidy more extensively in a separate study.

Reviewer #3 (Significance (Required)):

In their manuscript entitled: Crozier and co-workers studied the effects of CDK4/6 inhibition on cell growth. CDK4/6 inhibitors are currently used in the treatment for hormone-positive breast cancers, but their cell biological effects on tumor cells remain incompletely clear, which may hamper the further clinical development of these drugs for breast cancer or other cancers.

Inhibition of CDK4/6 is known to trigger a cell cycle arrest, and it is currently unclear how this could lead to long-term tumor control. This manuscript addresses the question why cdk4/6 inhibitors cause long-term cell cycle exit.

- We thank the reviewer for this simple description of our work, which we think pitches the significance very clearly. There are currently 18 different CDK4/6 inhibitors in clinical trials, and more than 100 further trials using the 3 currently licenced inhibitors in a wide variety of tumour types and

drug combinations. Although the clinical work on these drugs is huge, it is unclear how they cause long-term cell cycle arrest and we now link this to genotoxic stress for the first time. This explains clearly why this work is potentially very significant.

There are two issues that affect the significance of the findings:

-the authors start their manuscript with a strong translational/clinical issue, but solely use RPE1 cell lines to address this issue2. it remains unclear if their observations hold true in breast cancer models. it would be advised to repeat key findings in a hormone receptor-positive breast cancer model.

- We agree that the main caveat with our initial submission was the fact that it was unclear whether out findings were generally applicable to breast cancer cells. We have now performed extensive experiments at revision to address this issue. We now demonstrate that HR+ breast cancer cells treated with CDK4/6 inhibitor downregulate replisome components during G1, and then experience genotoxic damage when the cell cycle is resumed following drug washout (Figure 6). Importantly, we also demonstrate genotoxic damage in a variety of other tumour types, in this case during periods of constitutive drug exposure that does not cause a complete G1 arrest (figure 7). We believe this new data greatly enhances the significance of our findings.

-the effects of CDK4/6 inhibitors are observed in clinically relevant doses. however, the effects are observed upon switch-like wash out. this does not per se reflect the pharmacodynamics of more gradual increase and decrease of drug concentrations in tuner cells. by washing out the CDK4/6 inhibitors. the significant of this work would be greater if cell cycle exit with replication stress would be observed either in clinical samples or in vivo treated cancer cells.

- The new data added in other tumour types (figure 7) demonstrates that genotoxic damage can occur whilst cells are continually exposed to drug (i.e. without any drug washout periods). As we discuss at length in the new manuscript, we believe that this is likely to occur in patients in cells that cannot arrest permanently in G1 during the period of drug exposure. We agree, however, that the full significance of this work will ultimately only become apparent if replication stress is confirmed in clinical samples, and we feel it is now crucial to test this in breast cancer patients undergoing treatment. We feel that our work should stimulate exactly these kinds of studies.

- We would also like to add that the gradual increase/decrease in drug concentrations seen in patients is still likely to lead to switch like cell cycle re-entry given the switch-like nature of cell cycle controls at the G1/S transition. So, the timing may be different, but we predict that the downstream response in S-phase would remain the same. Therefore, it will also be crucial to address if replication stress is seen during drug-free washout periods in patients, as we also highlight in the discussion.

References

1. Tan, C., et al., *Cell size homeostasis is maintained by CDK4-dependent activation of p38 MAPK.* Dev Cell, 2021. **56**(12): p. 1756-1769.e7.

2. Patnaik, A., et al., *Efficacy and Safety of Abemaciclib, an Inhibitor of CDK4 and CDK6, for Patients with Breast Cancer, Non-Small Cell Lung Cancer, and Other Solid Tumors.* Cancer Discov, 2016. **6**(7): p. 740-53.
3. Wander, S.A., et al., *The genomic landscape of intrinsic and acquired resistance to cyclin-dependent kinase 4/6 inhibitors in patients with hormone receptor positive metastatic breast cancer.* Cancer Discov, 2020.
4. Ibarra, A., E. Schwob, and J. Méndez, *Excess MCM proteins protect human cells from replicative stress by licensing backup origins of replication.* Proc Natl Acad Sci U S A, 2008. **105**(26): p. 8956-61.
5. Ge, X.Q., D.A. Jackson, and J.J. Blow, *Dormant origins licensed by excess Mcm2-7 are required for human cells to survive replicative stress.* Genes Dev, 2007. **21**(24): p. 3331-41.
6. Méndez, J. and B. Stillman, *Chromatin association of human origin recognition complex, cdc6, and minichromosome maintenance proteins during the cell cycle: assembly of prereplication complexes in late mitosis.* Mol Cell Biol, 2000. **20**(22): p. 8602-12.
7. Matson, J.P., et al., *Rapid DNA replication origin licensing protects stem cell pluripotency.* Elife, 2017. **6**.
8. Whitfield, M.L., et al., *Identification of genes periodically expressed in the human cell cycle and their expression in tumors.* Mol Biol Cell, 2002. **13**(6): p. 1977-2000.
9. Leone, G., et al., *E2F3 activity is regulated during the cell cycle and is required for the induction of S phase.* Genes Dev, 1998. **12**(14): p. 2120-30.
10. Cook, J.G., et al., *Analysis of Cdc6 function in the assembly of mammalian prereplication complexes.* Proc Natl Acad Sci U S A, 2002. **99**(3): p. 1347-52.
11. Matson, J.P., et al., *Intrinsic checkpoint deficiency during cell cycle re-entry from quiescence.* J Cell Biol, 2019. **218**(7): p. 2169-2184.
12. O'Leary, B., et al., *The Genetic Landscape and Clonal Evolution of Breast Cancer Resistance to Palbociclib plus Fulvestrant in the PALOMA-3 Trial.* Cancer Discov, 2018. **8**(11): p. 1390-1403.
13. Costa, C., et al., *PTEN Loss Mediates Clinical Cross-Resistance to CDK4/6 and PI3Kα Inhibitors in Breast Cancer.* Cancer Discov, 2020. **10**(1): p. 72-85.
14. Li, Z., et al., *Loss of the FAT1 Tumor Suppressor Promotes Resistance to CDK4/6 Inhibitors via the Hippo Pathway.* Cancer Cell, 2018. **34**(6): p. 893-905.e8.
15. Condorelli, R., et al., *Polyclonal RB1 mutations and acquired resistance to CDK 4/6 inhibitors in patients with metastatic breast cancer.* Ann Oncol, 2018. **29**(3): p. 640-645.
16. Asghar, U.S., et al., *Single-Cell Dynamics Determines Response to CDK4/6 Inhibition in Triple-Negative Breast Cancer.* Clin Cancer Res, 2017. **23**(18): p. 5561-5572.
17. Liu, C.Y., et al., *Combination of palbociclib with enzalutamide shows in vitro activity in RB proficient and androgen receptor positive triple negative breast cancer cells.* PLoS One, 2017. **12**(12): p. e0189007.
18. Christenson, J.L., et al., *Activity of combined androgen receptor antagonism and cell cycle inhibition in androgen receptor-positive triple-negative breast cancer.* Mol Cancer Ther, 2021.
19. Klein, M.E., et al., *CDK4/6 Inhibitors: The Mechanism of Action May Not Be as Simple as Once Thought.* Cancer Cell, 2018. **34**(1): p. 9-20.
20. Prat, A., et al., *Ribociclib plus letrozole versus chemotherapy for postmenopausal women with hormone receptor-positive, HER2-negative, luminal B breast cancer (CORALLEEN): an open-label, multicentre, randomised, phase 2 trial.* Lancet Oncol, 2020. **21**(1): p. 33-43.
21. Hurvitz, S.A., et al., *Potent Cell-Cycle Inhibition and Upregulation of Immune Response with Abemaciclib and Anastrozole in neoMONARCH, Phase II Neoadjuvant Study in HR(+)/HER2(-) Breast Cancer.* Clin Cancer Res, 2020. **26**(3): p. 566-580.
22. Ma, C.X., et al., *NeoPalAna: Neoadjuvant Palbociclib, a Cyclin-Dependent Kinase 4/6 Inhibitor, and Anastrozole for Clinical Stage 2 or 3 Estrogen Receptor-Positive Breast Cancer.* Clin Cancer Res, 2017. **23**(15): p. 4055-4065.

23. Johnston, S., et al., *Randomized Phase II Study Evaluating Palbociclib in Addition to Letrozole as Neoadjuvant Therapy in Estrogen Receptor-Positive Early Breast Cancer: PALLET Trial.* J Clin Oncol, 2019. **37**(3): p. 178-189.
24. Roberts, P.J., et al., *Chemotherapy and CDK4/6 Inhibitors: Unexpected Bedfellows.* Mol Cancer Ther, 2020. **19**(8): p. 1575-1588.
25. Goel, S., et al., *CDK4/6 Inhibition in Cancer: Beyond Cell Cycle Arrest.* Trends Cell Biol, 2018. **28**(11): p. 911-925.
26. Knudsen, E.S. and A.K. Witkiewicz, *The Strange Case of CDK4/6 Inhibitors: Mechanisms, Resistance, and Combination Strategies.* Trends Cancer, 2017. **3**(1): p. 39-55.
27. Wagner, V. and J. Gil, *Senescence as a therapeutically relevant response to CDK4/6 inhibitors.* Oncogene, 2020.
28. Salvador-Barbero, B., et al., *CDK4/6 Inhibitors Impair Recovery from Cytotoxic Chemotherapy in Pancreatic Adenocarcinoma.* Cancer Cell, 2020. **37**(3): p. 340-353.e6.

Thank you for submitting your final revised manuscript for our consideration. It has now been positively re-reviewed by two of the original Review Commons referees (see comments below), and I am pleased to inform you that we have now accepted it for publication in The EMBO Journal.

REFEREE REPORTS

Referee #1:

The authors have thoroughly and thoughtfully addressed all my concerns. I congratulate them on a thorough and interesting piece of research and recommend publication without any further corrections on my part.

Sarah McClelland

Referee #3:

The authors have extensively addressed the comments, and have significantly improved the manuscript. I support publication of this study.

EMBO PRESS

YOU MUST COMPLETE ALL CELLS WITH A PINK BACKGROUND

PLEASE NOTE THAT THIS CHECKLIST WILL BE PUBLISHED ALONGSIDE YOUR PAPER

Corresponding Author Name: Adrian T Saurin

Journal Submitted to: EMBO J

Manuscript Number: EMBOJ-2021-108599R

Reporting Checklist For Life Sciences Articles (Rev. June 2017)

This checklist is used to ensure good reporting standards and to improve the reproducibility of published results. These guidelines are consistent with the Principles and Guidelines for Reporting Preclinical Research issued by the NIH in 2014. Please follow the journal's authorship guidelines in preparing your manuscript.

A- Figures
1. Data
The data shown in figures should satisfy the following conditions:

- the data were obtained and processed according to the field's best practice and are presented to reflect the results of the experiments in an accurate and unbiased manner.
- figure panels include only data points, measurements or observations that can be compared to each other in a scientifically meaningful way.
- graphs include clearly labeled error bars for independent experiments and sample sizes. Unless justified, error bars should not be shown for technical replicates.
- if n< 5, the individual data points from each experiment should be plotted and any statistical test employed should be justified
- Source Data should be included to report the data underlying graphs. Please follow the guidelines set out in the author ship guidelines on Data Presentation.

2. Captions

Each figure caption should contain the following information, for each panel where they are relevant:

- a specification of the experimental system investigated (eg cell line, species name).
- the assay(s) and method(s) used to carry out the reported observations and measurements
- an explicit mention of the biological and chemical entity(ies) that are being measured.
- an explicit mention of the biological and chemical entity(ies) that are altered/varied/perturbed in a controlled manner.

- the exact sample size (n) for each experimental group/condition, given as a number, not a range;
- a description of the sample collection allowing the reader to understand whether the samples represent technical or biological replicates (including how many animals, litters, cultures, etc.).
- a statement of how many times the experiment shown was independently replicated in the laboratory.
- definitions of statistical methods and measures:
  - common tests, such as t-test (please specify whether paired vs. unpaired), simple χ^2 tests, Wilcoxon and Mann-Whitney tests, can be unambiguously identified by name only, but more complex techniques should be described in the methods section;
  - are tests one-sided or two-sided?
  - are there adjustments for multiple comparisons?
  - exact statistical test results, e.g., P values = x but not P values < x;
  - definition of 'center values' as median or average;
  - definition of error bars as s.d. or s.e.m.

Any descriptions too long for the figure legend should be included in the methods section and/or with the source data.

In the pink boxes below, please ensure that the answers to the following questions are reported in the manuscript itself. Every question should be answered. If the question is not relevant to your research, please write NA (non applicable). We encourage you to include a specific subsection in the methods section for statistics, reagents, animal models and human subjects.

USEFUL LINKS FOR COMPLETING THIS FORM

http://www.antibodypedia.com
http://1degreebio.org
http://www.equator-network.org/reporting-guidelines/improving-bioscience-research-report

http://grants.nih.gov/grants/olaw/olaw.htm
http://www.mrc.ac.uk/Ourresearch/Ethicsresearchguidance/Useofanimals/index.htm
http://ClinicalTrials.gov
http://www.consort-statement.org
http://www.consort-statement.org/checklists/view/32-consort/66-title
http://www.equator-network.org/reporting-guidelines/reporting-recommendations-for-tumo
http://datadryad.org
http://figshare.com
http://www.ncbi.nlm.nih.gov/gap
http://www.ebi.ac.uk/ega
http://biomodels.net/
http://biomodels.net/miriam/
http://jjj.biochem.sun.ac.za
https://osp.od.nih.gov/biosafety-biosecurity-and-emerging-biotechnology/
http://www.selectagents.gov/

B- Statistics and general methods

Please fill out these boxes (Do not worry if you cannot see all your text once you press return)

1.a. How was the sample size chosen to ensure adequate power to detect a pre-specified effect size?	Based on previous experience with similar experiments
1.b. For animal studies, include a statement about sample size estimate even if no statistical methods were used.	NA
2. Describe inclusion/exclusion criteria if samples or animals were excluded from the analysis. Were the criteria pre-established?	For quantification of time-lapse images, if a cell disappeared from the field of view it was excluded as it could not be analysed. A different cell was analysed in these instances. This criterea was pre-establish before analysis. In replicate 3 of the proteomic analysis, almost all protein intensities in the 7 day palbociclib treatment group were zero, indicating improper TMT labelling. Therefore, this experimental group was excluded from further analysis (a note has been added to the methods to indicate this).
3. Were any steps taken to minimize the effects of subjective bias when allocating animals/samples to treatment (e.g. randomization procedure)? If yes, please describe.	To prevent bias in the analysis of all time-lapse imaging, 50 cells per confition were selected at the start of the movie before analysis commenced. For gH2AX and EdU tallies all cells were selected in the DAPI channel to prevent bias.
For animal studies, include a statement about randomization even if no randomization was used.	NA
4.a. Were any steps taken to minimize the effects of subjective bias during group allocation or/and when assessing results (e.g. blinding of the investigator)? If yes please describe.	No
4.b. For animal studies, include a statement about blinding even if no blinding was done	NA
5. For every figure, are statistical tests justified as appropriate?	Yes
Do the data meet the assumptions of the tests (e.g., normal distribution)? Describe any methods used to assess it.	NA

Is there an estimate of variation within each group of data?	No
Is the variance similar between the groups that are being statistically compared?	Yes

C- Reagents

6. To show that antibodies were profiled for use in the system under study (assay and species), provide a citation, catalog number and/or clone number, supplementary information or reference to an antibody validation profile. e.g., Antibodypedia (see link list at top right), 1DegreeBio (see link list at top right).	Done
7. Identify the source of cell lines and report if they were recently authenticated (e.g., by STR profiling) and tested for mycoplasma contamination.	Done

* for all hyperlinks, please see the table at the top right of the document

D- Animal Models

8. Report species, strain, gender, age of animals and genetic modification status where applicable. Please detail housing and husbandry conditions and the source of animals.	NA
9. For experiments involving live vertebrates, include a statement of compliance with ethical regulations and identify the committee(s) approving the experiments.	NA
10. We recommend consulting the ARRIVE guidelines (see link list at top right) (PLoS Biol. 8(6), e1000412, 2010) to ensure that other relevant aspects of animal studies are adequately reported. See author guidelines, under 'Reporting Guidelines'. See also: NIH (see link list at top right) and MRC (see link list at top right) recommendations. Please confirm compliance.	NA

E- Human Subjects

11. Identify the committee(s) approving the study protocol.	NA
12. Include a statement confirming that informed consent was obtained from all subjects and that the experiments conformed to the principles set out in the WMA Declaration of Helsinki and the Department of Health and Human Services Belmont Report.	NA
13. For publication of patient photos, include a statement confirming that consent to publish was obtained.	NA
14. Report any restrictions on the availability (and/or on the use) of human data or samples.	NA
15. Report the clinical trial registration number (at ClinicalTrials.gov or equivalent), where applicable.	NA
16. For phase II and III randomized controlled trials, please refer to the CONSORT flow diagram (see link list at top right) and submit the CONSORT checklist (see link list at top right) with your submission. See author guidelines, under 'Reporting Guidelines'. Please confirm you have submitted this list.	NA
17. For tumor marker prognostic studies, we recommend that you follow the REMARK reporting guidelines (see link list at top right). See author guidelines, under 'Reporting Guidelines'. Please confirm you have followed these guidelines.	NA

F- Data Accessibility

18: Provide a "Data Availability" section at the end of the Materials & Methods, listing the accession codes for data generated in this study and deposited in a public database (e.g. RNA-Seq data: Gene Expression Omnibus GSE39462, Proteomics data: PRIDE PXD000208 etc.) Please refer to our author guidelines for 'Data Deposition'.<br><br>Data deposition in a public repository is mandatory for:<br>a. Protein, DNA and RNA sequences<br>b. Macromolecular structures<br>c. Crystallographic data for small molecules<br>d. Functional genomics data<br>e. Proteomics and molecular interactions	Done
19. Deposition is strongly recommended for any datasets that are central and integral to the study; please consider the journal's data policy. If no structured public repository exists for a given data type, we encourage the provision of datasets in the manuscript as a Supplementary Document (see author guidelines under 'Expanded View' or in unstructured repositories such as Dryad (see link list at top right) or Figshare (see link list at top right).	All crucial data visible in graphical form
20. Access to human clinical and genomic datasets should be provided with as few restrictions as possible while respecting ethical obligations to the patients and relevant medical and legal issues. If practically possible and compatible with the individual consent agreement used in the study, such data should be deposited in one of the major public access-controlled repositories such as dbGAP (see link list at top right) or EGA (see link list at top right).	NA
21. Computational models that are central and integral to a study should be shared without restrictions and provided in a machine-readable form. The relevant accession numbers or links should be provided. When possible, standardized format (SBML, CellML) should be used instead of scripts (e.g. MATLAB). Authors are strongly encouraged to follow the MIRIAM guidelines (see link list at top right) and deposit their model in a public database such as Biomodels (see link list at top right) or JWS Online (see link list at top right). If computer source code is provided with the paper, it should be deposited in a public repository or included in supplementary information.	NA

G- Dual use research of concern

22. Could your study fall under dual use research restrictions? Please check biosecurity documents (see link list at top right) and list of select agents and toxins (APHIS/CDC) (see link list at top right). According to our biosecurity guidelines, provide a statement only if it could.	NA
